# Meeting report on the first Iranian congress of electrodiagnosis in peripheral nerve lesions

**DOI:** 10.1186/1749-7221-2-10

**Published:** 2007-04-14

**Authors:** Seyed M Rayegani, Hasan M Bahrami

**Affiliations:** 1 Physical Medicine & Rehabilitation Department, Shohada Medical Center, Shaheed Beheshti Medical University, Tehran, Iran

## Abstract

The Department of Physical Medicine, Rehabilitation and Electrodiagnosis of Shaheed Beheshti Medical University in collaboration with the Iranian Society of Physical Medicine and Rehabilitation (ISPMR) held the 1^st ^Congress of Electrodiagnostic Medicine in Peripheral Nerve Lesions on December 21–22, 2006. Electrodiagnostic medicine is a specific branch of medicine used by specialist physicians in the field of physical medicine and rehabilitation and/or neurology to diagnose, prognosticate and plan treatment options of peripheral nerve lesions. This meeting was hold to discuss multidisciplinary approaches to this common and important topic in the medical field.

## Background

Electrodiagnostic medicine is a specific branch of medicine used by specialist physicians in the field of physical medicine and rehabilitation and/or neurology to diagnose, prognosticate and plan treatment options of peripheral nerve lesions. This meeting was hold to discuss multidisciplinary approaches to this common and important topic in the medical field.

## Report

The 1st Congress of Electrodiagnostic Medicine in Peripheral Nerve Lesions was held in Tehran,Iran organized by the Department of Physical Medicine, Rehabilitation and Electrodiagnosis of Shaheed Beheshti Medical University in collaboration with the Iranian Society of Physical Medicine and Rehabilitation (ISPMR [1]) on 21 and 22 December, 2006.

The topics covered diagnosis, treatment (medical and surgical) and rehabilitation measures of peripheral nerve lesions. Twenty leaders in the field of peripheral nerve including: physiatrists, neurosurgeons, neurologists, orthopedists, hand surgeons, vascular surgeons, and plastic and reconstructive surgeons were invited as key lecturers to present the updates about peripheral nerves in upper and lower limbs. In addition to oral presentations, 2 panel discussions, "Carpal Tunnel Syndrome" and "Thoracic Outlet Syndrome," were organized to discuss these challenging topics. About 160 participitants from diverse disciplines including physiatry, orthopedics, neurosurgery, hand surgery, neurology, physiotherapy, occupational therapy and also general practitioners participated in the congress. Seyed Mansoor Rayegani, M.D., associate professor of PM&R, was responsible as the secretary of the congress.

The additional files contain the slides from the invited lectures and panel discussions [see additional file 1].

**Figure 1:**
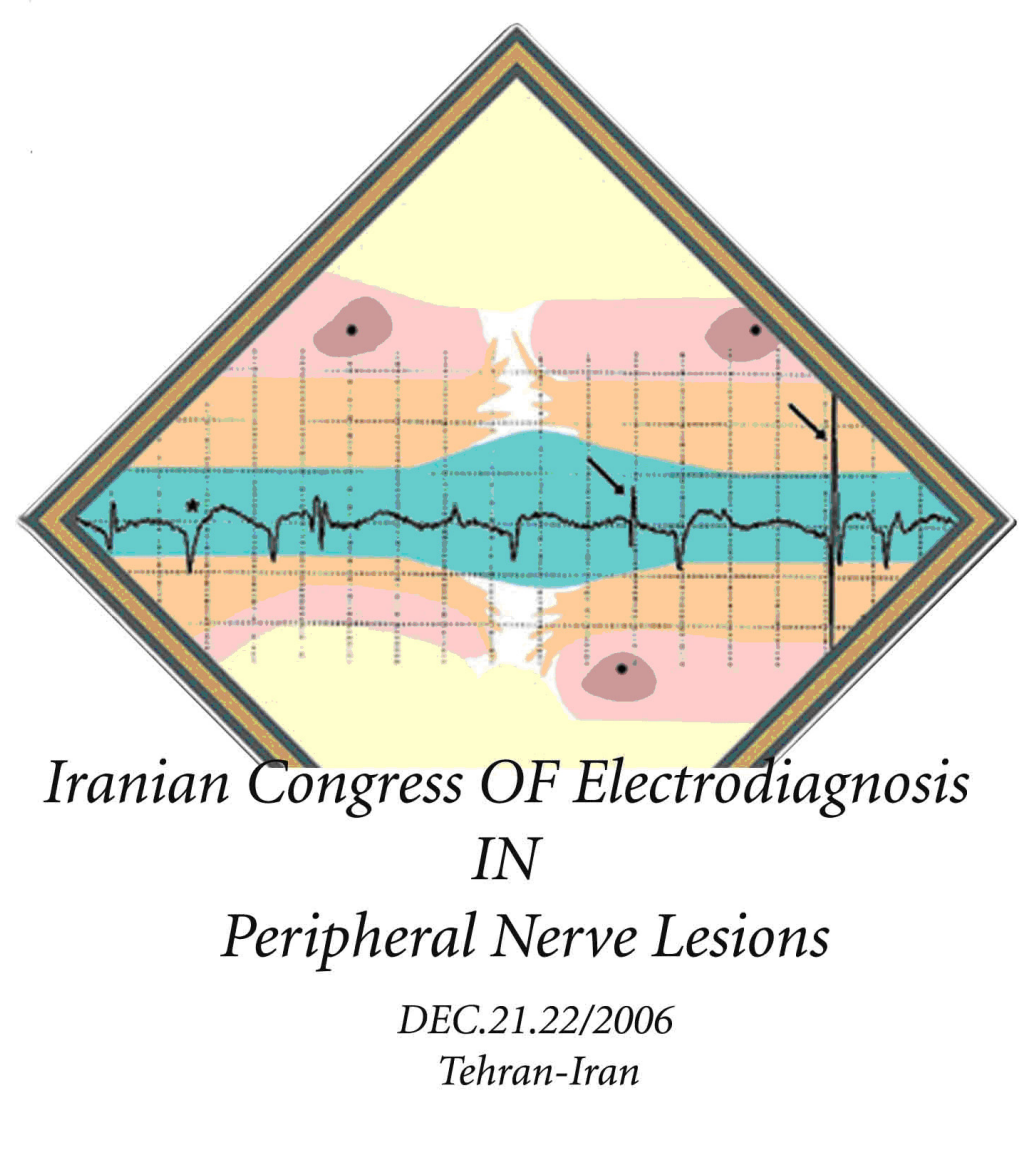
Logo of the conference.

**Figure 2:**
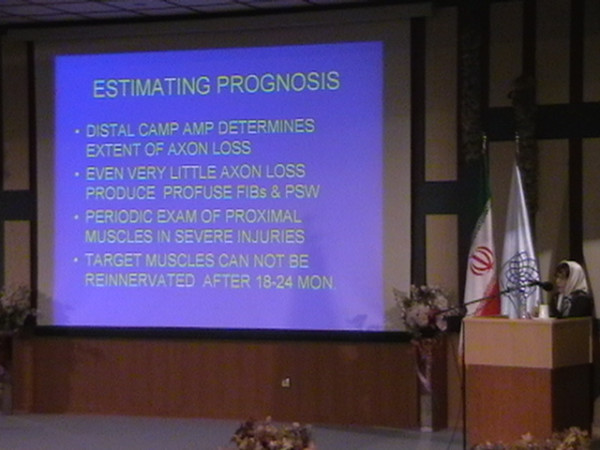
Dr M M Dehkorki presenting her lecture, titled Electrodiagnosis in Peripheral Nerve Lesions.

**Figure 3:**
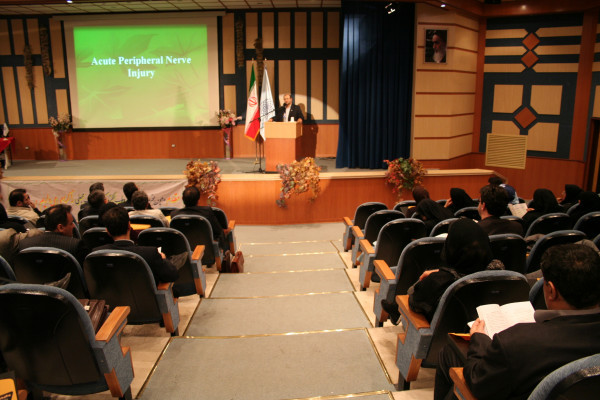
Dr A R Zalie presenting his lecture, titled Acute Peripheral Nerve Injury.

## Supplementary Material

Additional file 1 Slides from the invited lectures and panel discussions. Compressed PDFs of 15 presentations and 2 panel discussions during the conference. Click here for file
