# Supplementary material for: Meeting report on the first Iranian congress of electrodiagnosis in peripheral nerve lesions
Source: J Brachial Plex Peripher Nerve Inj. 2007 Apr 14;2:10. doi: 10.1186/1749-7221-2-10 (PMC1865540; doi:10.1186/1749-7221-2-10)
Supplement: Additional file 1 — Slides from the invited lectures and panel discussions. Compressed PDFs of 15 presentations and 2 panel discussions during the conference. [file 1749-7221-2-10-S1.zip › BASICS OF EDX IN PERIPHERAL NERVE LESIONS.pdf]

Iranian Congress OF Electrodiagnosis
IN
Peripheral Nerve Lesions
DEC.21.22/2006
Tehran-Iran

ELECTRODIAGNOSIS IN PERIPHERAL NERVE LESIONS

MANIGHEH MOHAMADPOOR DEHKORDI

M.D PHYSIATRIST

Iranian Congress OF Electrodiagnosis
IN
Peripheral Nerve Lesions
DEC.21.22/2006
Tehran-Iran

- ELECTRODIAGNOSTIC STUDIES:
- -LOCALIZING SITE OF INJURY
- -DETERMINING COMPLETE ,INCOMPLETE
- -PATHOPYYSIOLOGY
- -PROGNOSTIC INFORMATION
- -TIMING IS CRITICAL

Iranian Congress OF Electrodiagnosis
IN
Peripheral Nerve Lesions
DEC.21.22/2006
Tehran-Iran

AXONAL LESION

- -CLASSIFY COMPLETE ,INCOMPLETE
- -BEST INDEX: AMPLITUDE OF EVOKED CMAP ,SNAP
- -MAUP IN PARALYSED MUSCLE INDICATES PARTIAL LESION

EXTENT OF LESION

- -COMPLETE
- -INCOMPLETE

Iranian Congress OF Electrodiagnosis
IN
Peripheral Nerve Lesions
DEC.21.22/2006
Tehran-Iran

COMPLETE

- -ABSENT CAMPs
- -ABSENT SNAPs
- -NUMEROUS FIBRILLATIONS & POSITIVE SHARP WAVES

INCOMPLETE

- -SLOW MOTOR NCV, DECREASE AMPLITUDE OF CMAP
- -ABSENT SNAP OR DEC. NCV
- -MUPs WITH INC. POLY MUPs
- -FIBS & PSW

Iranian Congress OF Electrodiagnosis
IN
Peripheral Nerve Lesions
DEC.21.22/2006
Tehran-Iran

DEMYELINATION

- PRESERVED DISTAL CMAP AMP MORE THAN 7-10 DAYS
- -CONDUCTION BLOCK: COMPARE CMAP AMPs PROXIMAL & DISTAL
- -CV DECREASED ACROSS LESION
- NORMAL MUP, DEC. RECRUITMENT

Iranian Congress OF Electrodiagnosis
IN
Peripheral Nerve Lesions
DEC.21.22/2006
Tehran-Iran

NC. IN WALLERIAN DEG.

-SENS. COND. PRESERVED LONGER THAN MOTOR COND.

-LITTLE CHANGES IN NCV

-GRADUAL DECLINE IN AMP OF CAMPs AND SNAPs

-THE SHORTER DISTAL STUMP , THE EARLIER COND. FAILURE

TIMING OF EDX

- -BASELINE STUDY: 0-7 DAYS
- -INITIAL STUDY: 10-21 DAYS
- -FOLLOW UP STUDIES : 3-6 MONTHS
- -FOLLOWUP STUDIES : 6-12 MONTHS

REGENERATION & RECOVERY

- -3 MECHANISM :

- -REMYELINATION
- -AXONAL REGENERATION
- -COLLATERAL SPROUTING

Iranian Congress OF Electrodiagnosis
IN
Peripheral Nerve Lesions
DEC.21.22/2006
Tehran-Iran

REGENERATION

Iranian Congress OF Electrodiagnosis
IN
Peripheral Nerve Lesions
DEC.21.22/2006
Tehran-Iran

ANATOMICAL SEQUENCE

- -RECOVERY OF NEURONE
- -ONSET AT AXON TIP TO SITE OF INJ.
- -PASSAGE ACROSS THE ZONE OF INJ.
- -END ORGAN RELATIONSHIP
- -FUNCTIONAL MATURATION
- -COMPLETE RESTORATION OF FUNCTION

Iranian Congress OF Electrodiagnosis
IN
Peripheral Nerve Lesions
DEC.21.22/2006
Tehran-Iran

RATE OF REGENERATION

- -GENERALLY CONSTANT ,1 mm/day
- -PROXIMAL REG. 6-8 mm/day
- -DISTAL REG. 1-2 mm/day

Iranian Congress OF Electrodiagnosis
IN
Peripheral Nerve Lesions
DEC.21.22/2006
Tehran-Iran

EFFECTIVE FACTORS

-NATURE & LEVEL OF LESION

-GAP LENGTH

-EXTENT OF SCAR

-MAINTENANCE OF TARGET TISSUE

-AGE

-TEMP.

-INDIVIDUAL VARIATION

Iranian Congress OF Electrodiagnosis
IN
Peripheral Nerve Lesions
DEC.21.22/2006
Tehran-Iran

NCS UNDER REGENERATION

- -ELECTROPHYS. SENSORY FIBER RECOVERY POORER THAN MOTOR
- -SLOW NCV MAY PERSIST LONG AFTER REGEMERATION

Iranian Congress OF Electrodiagnosis
IN
Peripheral Nerve Lesions
DEC.21.22/2006
Tehran-Iran

RE-INNERVATION

- -GRADUAL DISAPPEARANCE OF FIBs
- -RETURNING MUPs (POLYs,WITH SHORT DURATION & SMALL AMP)
- -INCREASE RECRUITMENT
- -LARGE AMP LONG DURATION MUPs BY AXONAL SPROUTING

Iranian Congress OF Electrodiagnosis
IN
Peripheral Nerve Lesions
DEC.21.22/2006
Tehran-Iran

REMYELINATION

- -WITHIN 2 WEEKS OF ONSET OF REG.
- MYELINATED AXONS HAVE SHORTER INTERNODES

Iranian Congress OF Electrodiagnosis
IN
Peripheral Nerve Lesions
DEC.21.22/2006
Tehran-Iran

PROGNOSIS

- -PHYS.COND.BLOCK : EXCELLENT
- -NEURAPRAXIA RECOVERY WEEKS TO MONTHS
- AXONOTMESIS(TYPE 2) GOOD PROG.
- AXONOTMESIS(TYPE 3) POOR PROG.
- SURGERY MAY BE REQUIRED
- AXONOTMESIS (TYPE4) & NEUROTMESIS : SURGERY

Iranian Congress OF Electrodiagnosis
IN
Peripheral Nerve Lesions
DEC.21.22/2006
Tehran-Iran

ESTIMATING PROGNOSIS

- DISTAL CAMP AMP DETERMINES EXTENT OF AXON LOSS
- EVEN VERY LITTLE AXON LOSS PRODUCE PROFUSE FIBs & PSW
- PERIODIC EXAM OF PROXIMAL MUSCLES IN SEVERE INJURIES
- TARGET MUSCLES CAN NOT BE REINNERVATED AFTER 18-24 MON.

Iranian Congress OF Electrodiagnosis
IN
Peripheral Nerve Lesions
DEC.21.22/2006
Tehran-Iran

INPORTANT NOTES

- NO ELECTROPHYS. PARAMETER TO DISTINGUISH BETWEEN AXONOTMESIS & NEUROTMESIS AND BETWEEN CONTINUITY AND DISCONTINUITY OF A DAMAGED NERVE
