# Supplementary material for: Meeting report on the first Iranian congress of electrodiagnosis in peripheral nerve lesions
Source: J Brachial Plex Peripher Nerve Inj. 2007 Apr 14;2:10. doi: 10.1186/1749-7221-2-10 (PMC1865540; doi:10.1186/1749-7221-2-10)
Supplement: Additional file 1 — Slides from the invited lectures and panel discussions. Compressed PDFs of 15 presentations and 2 panel discussions during the conference. [file 1749-7221-2-10-S1.zip › BRACHIAL PLEXOPATHY.pdf]

Iranian Congress OF Electrodiagnosis
IN
Peripheral Nerve Lesions
DEC.21.22/2006
Tehran-Iran

Brachial Plexus Birth Palsy

- **Perinatal Risk Factures:**
  - Macrosomia
  - Multiparous Pregnancies
  - Previous Brachial Plexus Palsy
  - Difficult & Assisted delivery (Vacuum or Forceps)
  - Breech Delivery

Iranian Congress OF Electrodiagnosis
IN
Peripheral Nerve Lesions
DEC.21.22/2006
Tehran-Iran

Types

- *Upper Trunk (C_5- C_6), Most Common*
- *Upper Trunk with C_7 Injury*
- *Entire Plexus (C_5- T_1)*
- *Lower Trunk- C_8- T_1 (Extremely rare)*

Iranian Congress OF Electrodiagnosis
IN
Peripheral Nerve Lesions
DEC.21.22/2006
Tehran-Iran

Pathology

- *Neuroapraxia (Sunderland I)*
- *Axonotmesis (Sunderland II- IV)*
- *Neurotmesis (V)*
- *Avulsion*

Mechanically

- *Stretch (S. I)*
- *Ruptures (S. II- V)*
- *Avulsions*

Iranian Congress OF Electrodiagnosis
IN
Peripheral Nerve Lesions
DEC.21.22/2006
Tehran-Iran

Level

- **Preganglionic (Avulsions):**
  - *Lower plexus is most common, less C5- C6 will not recover spontaneously. Micro surgery before 3 months.*
- **Postganglionic:**
  - *Recovery key indictor is biceos function up to 3 months, other wise, reconstruction with never graft confrovers & surgery.*

Iranian Congress OF Electrodiagnosis
IN
Peripheral Nerve Lesions
DEC.21.22/2006
Tehran-Iran

Distinguishing of avulsion & extra forminal ruptures

- *Myelography- CT myelography- MRI*
- *Final decision for presence of avulsion is intra operatively.*

Iranian Congress OF Electrodiagnosis
IN
Peripheral Nerve Lesions
DEC.21.22/2006
Tehran-Iran

- ***Electra Diagnosis:***
  - *Diagnosis of severity of neural lesion.*
  - *Normal sensory (SNAP) & absent motor (CMAP) indicates avulsion.*
  - *Absence of reinnervation at 3 months indicates avulsion.*
- ***EMG:***
  - *Motor unit activity is not accurate in predicting level & degree of injury & regeneration.*
  - *So, most rely on physical exam for recovery & time of operation.*

Iranian Congress OF Electrodiagnosis
IN
Peripheral Nerve Lesions
DEC.21.22/2006
Tehran-Iran

Rehabilitation Goals

- *Prevent join contractures.*
- *Strengthening recovering muscles.*
- *Achieve developmental mile stones.*
- *Monitor recovery for surgical & non surgical management.*

Iranian Congress OF Electrodiagnosis
IN
Peripheral Nerve Lesions
DEC.21.22/2006
Tehran-Iran

Prognosis

- *Majority is transient.*
- *Who recovers partial antigravity upper trunk muscle strength in the 1st 2 months, have complete recovery in 1-2 years.*
- *Who dose recover antigravity biceps strength by 5-6 months, should have microsurgical reconstruction.*
- *Partial recovery of C_5 to C_7 antigravity strength during month 3-6 will have permanent weakness & LOM.*

Nerve surgery

1) Neurolysis
2) Nerve resection & grafting
3) Nerve transfers
    1) *Direct nerve is rarely performed*

Iranian Congress OF Electrodiagnosis
IN
Peripheral Nerve Lesions
DEC.21.22/2006
Tehran-Iran

Neurolysis

- *No role in avulsion (some: no role at all)*

Some:

- *By intra operative EMG: By more than 50% of CMAP across neuroma, then neurolysis can be performed other wise resection of neuroma & graft.*

Iranian Congress OF Electrodiagnosis
IN
Peripheral Nerve Lesions
DEC.21.22/2006
Tehran-Iran

- *Neuroma resection & graft:*
  - *In extra forminal rupture.*
- *Nerve transfer (+grafting):*
  - *In segmental or total avulsions.*
