# Supplementary material for: Meeting report on the first Iranian congress of electrodiagnosis in peripheral nerve lesions
Source: J Brachial Plex Peripher Nerve Inj. 2007 Apr 14;2:10. doi: 10.1186/1749-7221-2-10 (PMC1865540; doi:10.1186/1749-7221-2-10)
Supplement: Additional file 1 — Slides from the invited lectures and panel discussions. Compressed PDFs of 15 presentations and 2 panel discussions during the conference. [file 1749-7221-2-10-S1.zip › CPN LESIONS.pdf]

Iranian Congress OF Electrodiagnosis
IN
Peripheral Nerve Lesions
DEC.21.22/2006
Tehran-Iran

IN THE NAME OF GOD

K.Azma physiatrist

Army university of medical science

Iranian Congress OF Electrodiagnosis
IN
Peripheral Nerve Lesions
DEC.21.22/2006
Tehran-Iran

Electrodiagnosis
in
proneal N injury

Iranian Congress OF Electrodiagnosis
IN
Peripheral Nerve Lesions
DEC.21.22/2006
Tehran-Iran

Anatomy

- short head of the biceps femoris, the only peroneal-derived muscle above the levelof the fibular neck
- lateral cutaneous nerve of the knee, which supplies sensation to the lateral knee before winding around the fibular neck
- In 15-20% of patients, an accessory peroneal nerve leaves the main superficial peroneal nerve and runs posterior to the lateral malleolus to ultimately supply the lateral EDBmuscle.

Causes of proneal neuropathy

- Acute peroneal neuropathy often follows trauma, forcible stretch injury, or compression from prolonged immobilization. In the hospital, this occurs most often postoperatively in patients who have received anesthesia or heavy sedation.
- Slowly progressive lesions often suggest a mass lesion, such as a ganglion or nerve sheath tumor.

causes

- In addition, patients who have recently lost a substantial amount of weight may be prone to peroneal palsy, probably because of the lack of protective supporting adipose tissue at the fibular neck.

Physical Examination in proneal n. lesion :

- ankle inversion is spared, mediated by the tibialis posterior (L5, sciatic-tibial nerve).
- If the ankle is tested in a dropped position, however, ankle inversion may appear weak (similar to testing finger abduction in a dropped wrist position).
- Accordingly, to test ankle inversion in a patient with a footdrop, the ankle should be passively dorsiflexed to avoid the mistaken impression that the tibialis posterior is weake

Iranian Congress OF Electrodiagnosis
IN
Peripheral Nerve Lesions
DEC.21.22/2006
Tehran-Iran

P/H in proneal N injury

- Sensation is normal over the lateral foot (sural territory), sole of the foot (medial and lateral plantar territory), and medial calf and foot (saphenous territory).
- Sensation over the lateral knee is also preserved because that area is innervated by the lateral cutaneous nerve of the knee, which arises from the common peroneal nerve above the fibular neck.

E in proneal N. Injury

- all reflexes, including the ankle reflex, remain normal in an isolated peroneal neuropathy.

P/E in lesions proximal to proneal N at the fibular Neck

- 1. Weakness of ankle inversion (tibialis posterior)
- 2. Preferential weakness of the EHL (L5-S1) out of proportion to the TA (L4-L5), when compared with one another. (In a peroneal neuropathy, these two muscles are usually equally affected; in an L5 radiculopathy, the EHL is usually weaker than the TA because of its predominant L5 innervation.)
- 3. Sensory loss over the lateral knee (distribution of the lateral cutaneous nerve of the knee)

P/E in lesions proximal to proneal N at the fibular Neck

- 4. Sensory loss over the sole of the foot, lateral foot, or medial calf (distribution of the plantar, sural, or saphenous nerves, respectively)
- 5. Any weakness of hip abduction, extension, or intemal rotation (gluteus medius, gluteus maximus, tensor fascia latae).
  (Because these muscles are quite strong, they must be tested at mechanical disadvantage to demonstrate subtle weakness.)
- 6. Any asymmetry of the ankle reflex

- lesions of the sciatic nerve, lesions of the lumbosacral plexus, and L5 radiculopathy may occasionally mimic a peroneal palsy almost exactly, including abnormalities of sensation. It is in these cases that electrical studies are especially helpful.

In addition, the electrophysiologic evaluation can usually

- localize the level of theperoneal neuropathy,
- identify the underlying pathophysiology
- \ establish the prognosis.

Recommended NCS Protocol for Peroneal N lesion Routine studies:

- 1. Peroneal motor study, recording EDB, stimulating ankle, below fibular neck and lateral popliteal fossa (If there is no focal slowing or conduction block at the fibular neck, perform a peroneal motor study, recording tibialis anterior and stimulating below the fibular neck and lateral popliteal fossa.)
- …………………..

Iranian Congress OF Electrodiagnosis
IN
Peripheral Nerve Lesions
DEC.21.22/2006
Tehran-Iran

NCS Protocol

- 2. Tibial motor study, recording abductor hallucis brevis, stimulating medial ankle and popliteal fossa
- 3. Superficial peroneal sensory study, stimulating lateral calf, recording lateral ankle
- 4. Sural sensory study, stimulating calf, recording posterior ankle
- 5. Tibial and peroneal F responses

Special consideration :

- If any study is abnormal or borderline, especially the motor or sensory amplitudes, comparison to the contralateral asymptomatic side is often useful

Electrophysiologic finding :

- SNAP of SPN ;
- Loss of AMP (50%)in Axonal type lesion of CPN & SPN
- ccurrance of a reduced sensory response of SPN due to L5 radiculopathy is described

Iranian Congress OF Electrodiagnosis
IN
Peripheral Nerve Lesions
DEC.21.22/2006
Tehran-Iran

Recommended Electromyographic Protocol for Peroneal Neuropathy

Routine muscles:

- 1. At least two muscles innervated by the deep peroneal nerve (e.g., tibialis anterior, extensor hallucis longus)
- 2. At least one muscle innervated by the superficial peroneal nerve (e.g., peroneus longus, peroneus brevis)
- 3. Tibialis posterior and at least one other tibial muscle (e.g., medial gastrocnemius, soleus, flexor digitorum longus)
- 4. Short head of the biceps femoris

exclude a superimposed lesion

- if the conduction studies localize the lesion to the peroneal nerve at the fibular neck (focal slowing or conduction block) a few critical non-peroneal-innervated L5 muscles should still be sampled to confirm that the lesion is indeed restricted to the peroneal nerve and to exclude a superimposed lesion

Tibialis postrior & hamstring muscle

Special Consideration :

- • If the short head ofthe biceps femoris or any tibial innervated muscle is abnormal
- if nerve conduction studies demonstrate a nonlocalizing peroneal neuropathy
- if abnormal tibial motor or sural responses

a more extensive needle examination of other sciatic, gluteal, and paraspinal muscles should be performed to identify the level of the lesion.

Iranian Congress OF Electrodiagnosis
IN
Peripheral Nerve Lesions
DEC.21.22/2006
Tehran-Iran

Usefulness of recording the tibialis anterior in peroneal neuropathy

- recording the tibialis anterior is often more informative than routine studies recording the extensor digitorum brevis. in patients with a foot drop, it is weakness of the TA that accounts for the clinical deficit
- In some cases of peroneal neuropathy at the fibular neck, conduction block may be seen recording the tibialis anterior but not the extensor digitorum brevis.

EMG of short head of biceps :

- Manal muscle testing for this muscle is limited
- In some cases, sciatic neuropathy may mimic the EMG pattern of peroneal neuropathy, with the exception of abnormalities found in the short head of the biceps femoris.
- Abnormalities in this muscle or in any of the hamstring muscles imply a lesion proximal to the peroneal nerve, in the sciatic nerve or higher.

Thanks
