# Supplementary material for: Meeting report on the first Iranian congress of electrodiagnosis in peripheral nerve lesions
Source: J Brachial Plex Peripher Nerve Inj. 2007 Apr 14;2:10. doi: 10.1186/1749-7221-2-10 (PMC1865540; doi:10.1186/1749-7221-2-10)
Supplement: Additional file 1 — Slides from the invited lectures and panel discussions. Compressed PDFs of 15 presentations and 2 panel discussions during the conference. [file 1749-7221-2-10-S1.zip › CTS.pdf]

Iranian Congress OF Electrodiagnosis
IN
Peripheral Nerve Lesions
DEC.21.22/2006
Tehran-Iran

Electrodiagnostic Medicine

in

Carpal Tunnel Syndrome

Seyed mansoor rayegani M.D

Associate professor

of

Physical medicine & rehabilitation

Shohada medical center

Shaheed beheshti medical university

Iranian Congress OF Electrodiagnosis
IN
Peripheral Nerve Lesions
DEC.21.22/2006
Tehran-Iran

Definition

Compressive neuropathy of median nerve at carpal tunnel **Causing** pain,numbness,tingling and weakness in median nerve territory.

Clinical manifestation

- **Up to 2.7 % of Americans are involved**
- **M/F 3/1 TO 10/1**
- **Bilateral 50 to 90%**

Early (sensory complaints mainly at night, positive clinical tests ,no motor deficit)

Intermediate (+ drop objects , sensory complaints at daytime, some motor involvement)

Advanced(++ advanced motor involvement with thenar atrophy)

Iranian Congress OF Electrodiagnosis
IN
Peripheral Nerve Lesions
DEC.21.22/2006
Tehran-Iran

Clinical tests

- Phalen test
- Durkan test
- Tinle test
- Tourniquet test
- Median stress test
- Monofilament
- 2-ponit discrimination

Very diverse clinical appraisal of tests by clinicians

(am j ind. Med. 2006 jan 49(1):8-12)

Diagnostic tests

Anatomic

- MRI:around $1,000/test: to estimate severity of CTS: not used routinely but is capable of detecting abnormalities indicative of CTS.

- Sonography
- CT-Scan

Physiologic

Electrodiagnosis

Portable device

(Primary diagnosis , prognosis , dif. Diagnosis)

Iranian Congress OF Electrodiagnosis
IN
Peripheral Nerve Lesions
DEC.21.22/2006
Tehran-Iran

Electrodiagnosis

Extension of complete ,relevant medical examination including history taking, pertinent physical exam. ,imaging and lab tests review

Sensory & motor NCS

- **Latency (the most important)**
- **Amplitude (prognosis indicator & alarming for proximal lesions)**
- **NCV(across wrist, forearm??)**
- **F-wave???**

EMG

- **Axonal damage evaluation**
- **other lesions rather than CTS**
- ***NO ROLE IN EARLY DIAGNOSIS***

Iranian Congress OF Electrodiagnosis
IN
Peripheral Nerve Lesions
DEC.21.22/2006
Tehran-Iran

Nerve conduction study

(motor & sensory)

- Segmental median sensory NCS
  - Absolute latency(3rd digit 14cm antiodromic method)
  - Across wrist: stimulation at palm 7cm
  - CNAP: orthodromic stimulation at 8cm
  - Inching :segmental localization
- Comparative sensory ncs
  - With ulnar: by 5th digit and 4th digit
  - With radial: bactrian sign
  - CSI:<0.9ms

Iranian Congress OF Electrodiagnosis
IN
Peripheral Nerve Lesions
DEC.21.22/2006
Tehran-Iran

Motor NCS

- **Absolute**

  Thenar by 8cm stimulation

- **Comparative**

  Ulnar :hypothenar,thenar,lumbrical/interosseous

National clearinghouse guidelines

National Guideline Clearinghouse
www.guideline.gov

Practice standards: generally accepted principles for patient management that reflect a high degree of clinical certainty.

Practice guidelines: recommendations for patient management that reflect moderate clinical certainty.

Practice options: other strategies for patient management for which the clinical utility is uncertain.

STANDARD

1-Median sensory latency 3rd digit antidromic compare with ulnar and radial if the result was abnormal

2-if 1 is normal Do:

- **median CNAP at wrist 8cm orthodromic and compare with ulnar CNAPat the same way**
- **median/ulnar/radial sensory at 4th and 1st digits**
- **segmental comparison of wrist segment sensory median latency with digit or forearm**

Iranian Congress OF Electrodiagnosis
IN
Peripheral Nerve Lesions
DEC.21.22/2006
Tehran-Iran

Guideline

Median motor NCS at wrist with comparison to ulnar at wrist

Optional

- **Median /ulnar motor comparison via 2nd lumbrical /2nd interosseous**
- **Median motor latency at palm and wrist**
- **Median motor amplitude at wrist and palm**
- **Inching methods**
- **Needle EMG of thenar and C5 to T1 myotomes**

Iranian Congress OF Electrodiagnosis
IN
Peripheral Nerve Lesions
DEC.21.22/2006
Tehran-Iran

National Guideline Clearinghouse
www.guideline.gov

NOT recommended

- F-wave
- SSR
- NCS with limb ischemia
- NCS with dynamic hand exercise
- NCS with wrist positioning

Electrodiagnostic severity index

Mild: Sensory involvement only

Moderate: + motor prolongation

Severe: + + denervation in thenar muscles.
