# Supplementary material for: Meeting report on the first Iranian congress of electrodiagnosis in peripheral nerve lesions
Source: J Brachial Plex Peripher Nerve Inj. 2007 Apr 14;2:10. doi: 10.1186/1749-7221-2-10 (PMC1865540; doi:10.1186/1749-7221-2-10)
Supplement: Additional file 1 — Slides from the invited lectures and panel discussions. Compressed PDFs of 15 presentations and 2 panel discussions during the conference. [file 1749-7221-2-10-S1.zip › E_LECTRODIAGNOSTIC PROGNOSTICATION OF NERVE LESIONS.pdf]

Iranian Congress OF Electrodiagnosis
IN
Peripheral Nerve Lesions
DEC.21.22/2006
Tehran-Iran

Electrodiagnostic prognostication

By S. Khosrawi MD

IUMS , Isfahan , Iran

Iranian Congress OF Electrodiagnosis
IN
Peripheral Nerve Lesions
DEC.21.22/2006
Tehran-Iran

The purposes :

- To clarify some aspects and limitations of prognostication value of EDX testing
- Describe Seddon's and Sunderland's models for determination of the degree of nerve fiber injury
- Present some articles(anesthesia-related nerve injuries, Evaluation of facial palsy, vocal fold paralysis, GBS, peroneal neuropathy, Disorders of Consciousness,....)

- **Electrodiagnostic testing** encompasses **two roles** in the evaluation and management of patients with neuromuscular disease :

✓ 1 * Most frequently, EDX testing is used as an extension of the neurological examination. Like any of the other building blocks of the medical and neurological history and examination sequence, EDX testing can……assist in making a diagnosis
while………………..excluding alternatives,
assist in……………..patient education,
provide……………..**prognostic information**,
and can……………...guide and monitor treatment.

- ✓ 2 * However, EDX testing may also be **considered as a test** , with a different status than that of the clinical examination. Viewed in this way, validation of its utility in making a diagnosis requires using a **reference standard case definition** that is derived independently of the results of the EDX test themselves.
This is a relatively recent distinction.

Iranian Congress OF Electrodiagnosis
IN
Peripheral Nerve Lesions
DEC.21.22/2006
Tehran-Iran

Several models allow for a clinical determination of the degree of nerve fiber injury :

- **Seddon** originally proposed that peripheral nerve injury involves varying degrees of

  neurapraxia (blockade) ,

  axonotmesis (division of individual fibers), and **neurotmesis** (division of fascicles and epineurium)

- **Sunderland** produced a clinical–pathologic classification of nerve injury that expanded on Seddon’s pathologic scheme and grades the extent of injury as follows:

First-degree injury is characterized by blockage of axoplasm flow within the axon. This blockade constitutes a *neuropraxic injury* according to Seddon’s classification.

Second-degree injuries entail axonal and myelin disruption distal to the injury site as a result of progression of a first-degree injury.

Third-degree injuries involve complete disruption of the axon and its surrounding myelin and endoneurium.

Fourth-degree injuries entail complete disruption of the perineurium.

Fifth-degree injuries entail disruption of the epineurium.

Iranian Congress OF Electrodiagnosis
IN
Peripheral Nerve Lesions
DEC.21.22/2006
Tehran-Iran

Classification of peripheral nervous lesions:

Suderland	*1st degree*	*2nd degree*	*3rd degree*	*4th degree*	*5th degree*
Seddon	Neurapraxia	Axonotmesis	Axonotmesis	Axonotmesis	Neurotmesis
EDx	Conduction block	Axonal loss			
Pathology	Demyelin.	Axon & myelin. Intact supporting	Disrupted endoneureum	Disrupted endo. and perineurium	All supporting structures (inc. epineurium)
Prognosis	2-3 mos. excellent recovery	Slow recovery	Protracted	unlikely without surgery	Impossible without surgery

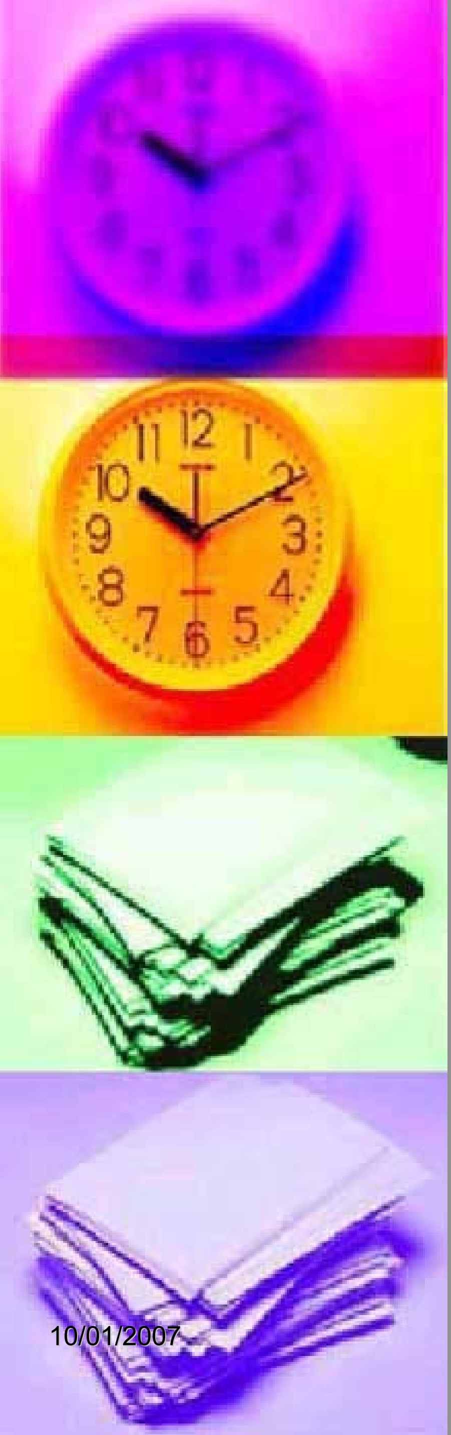

Axon loss (Wallerian degeneration)

- EDX abnormalities :

**Distal CMAP:**	**5-6**	**days**
**Distal SNAP:**	**10-11**	**days**
**Fibrillation and PSWs:**	**14-21**	**days**

Regeneration

- "nascent" potentials within 2 months

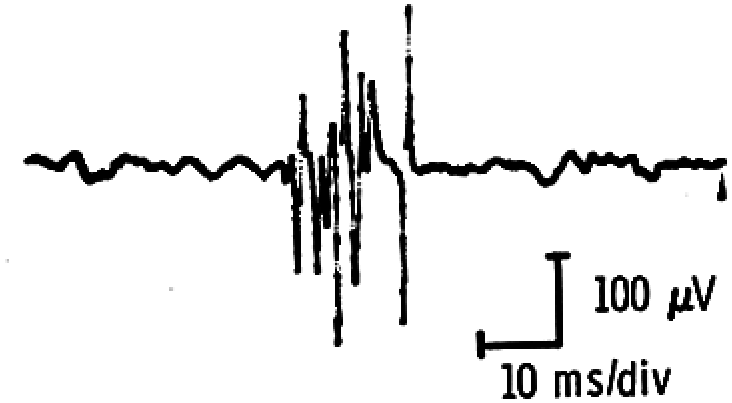

- Within 4-6 months the motor units become of longer duration, of higher amplitude, and are less polyphasic.
- At about 8-12 months they reach normal size for the muscle being examined.

Iranian Congress OF Electrodiagnosis
IN
Peripheral Nerve Lesions
DEC.21.22/2006
Tehran-Iran

Reinnervation

- reinnervation is accomplished by **collateral sprouting** ,begins in the **first two months** after nerve injury and results in prolongation of the reinnervating motor unit duration and an increase in the number of its phases

- **Sunderland** model predicts a high likelihood for **complete recovery** of peripheral innervation when endoneurial tubules remain intact to support reinnervation, as is the case with **first- and second-degree injuries**.
- In contrast, disruption of the endoneurium (a **third degree injury or worse**) increases the likelihood of **irreversible axonal injury and aberrant patterns of regeneration**.
- Correlation of the ultimate level of recovery with early electrophysiologic findings determines the test's **prognostic value**.

Iranian Congress OF Electrodiagnosis
IN
Peripheral Nerve Lesions
DEC.21.22/2006
Tehran-Iran

Evaluation of facial palsy

- **Histopathologic studies of Bell’s palsy and herpes zoster oticus suggest that nerve injuries as severe as fourth-degree injury may occur in these pathologies.**

Evaluation of facial palsy

- **Single *most important prognostic factor* is : whether the palsy is *complete or incomplete ?***
- ***incomplete*** **palsies have satisfactory recovery of facial function;**
- ***complete*** **paralysis often portends a poorer prognosis, and requires quicker assessment and management.**
- **Electrophysiologic tests (NET, MST, ENoG, EMG): helps determine endpoint of degeneration and prognosis for return of motor function, usually in preparation for surgery.**

Nerve excitability test:

The lowest electric current (threshold) necessary to elicit a facial twitch on the paralyzed side is compared with the threshold value of the "normal“ (contralateral) side.

A *difference of more than 3.5 milliamperes* between sides portends a poor prognosis for return of facial function;

**NOT RELIABLE !*

Maximal stimulation test:

(modified form of NET)

"patients' tolerance"

Graded as : (1) equal to the contralateral side, (2) minimally diminished (>50% of normal), (3) markedly diminished (< 25% of normal), or (4) absent.

Latter two grades (within the first 2 weeks of the clinical paralysis) suggest advanced degeneration and a poor prognosis(75% chance of incomplete facial nerve recovery)

Conversely, if responses were symmetric during the first 2 weeks of a clinical paralysis, complete return was found in more than 90% of patients tested.

**SUPERIOR TO NET !*

Evoked ElectroMyoGraphy (EEMG) or Electroneurography(ENoG)

EEMG (ENoG) :

The peak-to-peak amplitude is compared to the amplitude of the normal side.

Greater than 90% degeneration compared to the normal side indicates a poor prognosis.

When results demonstrated less than 90% denervation (10% in CMAP amplitude relative to the normal side), *excellent recovery* was uniformly observed.

Evoked electromyography testing should be repeated on an every-other-day basis to detect ongoing degeneration beyond the 90% critical level. The earlier the EEMG response drops to 10% or less than normal, the worse the prognosis.

Facial EEMG (ENoG) is most reliable only during the first 2–3 weeks (especially on the fifth day) following onset of a paralysis

Considered to be an accurate prognostic test ; EEMG (ENoG) is now recognized as *the most accurate* and straightforward means of assessing the degree of nerve injury

- During the first three weeks, Greater than 90% degeneration compared to the normal side indicates a poor prognosis and that patient should be explored.
- Anytime after three weeks, there is some controversy regarding the decision to explore, primarily because some nonpenetrating injuries can show spontaneous recovery.
- Patients with clinically incomplete paralyses due to Bell’s palsy invariably recover function to normal or near-normal levels and do not require EEMG evaluation.
- The reappearance of facial movement within 3–4 weeks after onset also predicts an excellent prognosis for functional recovery.

Electromyography:

- May be complementary to NET, MST, and ENoG during the first 3-4 days after injury. If voluntary active motor units are present, nerve is probably intact with incomplete injury, but if active motor are not present, there is some undetermined degree of facial nerve injury because EMG's cannot differentiate totally neuropraxic nerve from a completely degenerated one.
- Polyphasic potentials are seen during nerve regeneration and may be seen as early as 4-6 weeks after onset of paralysis.

Nerve conduction time (latency) :

Upper limits of normal:

adults: 4 msec ,

neonates/infants: 7-10 msec.;

The least reliable prognostic test !

Iranian Congress OF Electrodiagnosis
IN
Peripheral Nerve Lesions
DEC.21.22/2006
Tehran-Iran

* *Don't forget :*

NET, MST, **ENoG :**

- **are most useful in evaluating acute paralysis while nerve is in degenerative phase.**
- **show normal results for the first 3-4 days after nerve injury.**
- **will only work if the patient has unilateral involvement since the tests are predicated on the existence of a "normal" contralateral side for comparison.**

Trigeminal blink reflex:

Kimura et al.:

An abolished R1 response was associated with little chance of recovery in the first 2 months following the onset of paralysis.

Preserved R1 responses early on predicted return within the first month.

Only prognostic test that measures central pathology of facial nerve.

*** Considered investigational ***

Arch Otolaryngol Head Neck Surg.
2001;127:155-160.

- **Objective** To analyze the value of electromyography in predicting recovery from acute **neurogenic vocal fold** paralysis.
- **Intervention** Prognostication was based on electromyography performed no earlier than 14 days after onset of palsy. Findings were classified as neurapraxy, axonotmesis, and neurotmesis.
- **Results** By means of laryngeal electromyography, defective recovery, defined as absence of completely free vocal fold mobility, was predicted correctly in 94.4% of cases (68/72). For complete recovery, prognosis was accurate in only 12.8% of cases (5/39).
- **Conclusions** The **detection of neural degeneration** by laryngeal electromyography **allows the prediction of poor functional outcome with sufficient reliability in an early phase of the disease process.** Conversely, the absence of signs of degeneration does not imply that complete recovery is to be expected.

Iranian Congress OF Electrodiagnosis
IN
Peripheral Nerve Lesions
DEC.21.22/2006
Tehran-Iran

S (North American Plasmapheresis Trial) :

- 4 independent factors useful for **prognostication**

1) age;
2) need for mechanical ventilation;
3) need for plasmapheresis; and
4) mean distal CMAP amplitude less than 20% the normal lower limit.

Iranian Congress OF Electrodiagnosis
IN
Peripheral Nerve Lesions
DEC.21.22/2006
Tehran-Iran

Aminoff, Accepted for publication September , 2003

- The purpose of the article is to clarify the use and limitations of electrophysiologic testing in the diagnosis and management of anesthesia-related nerve injuries, ulnar nerve (28%) ,brachial plexus (20%).
- electrophysiologic testing is important in defining the neurogenic basis of weakness and localizing the site of the lesion, determining the severity of injury and thus in guiding prognostication.
- The electromyographic findings may provide a guide to the time of onset of the lesion and to its chronicity, and this may have medicolegal implications.
- More information is provided if the examination is repeated approximately 4 weeks after injury
- Serial studies are generally not required because progress can be followed clinically.

Iranian Congress OF Electrodiagnosis
IN
Peripheral Nerve Lesions
DEC.21.22/2006
Tehran-Iran

Usefulness of EDX in peroneal neuropathy by American Association of Neuromuscular & Electrodiagnostic Medicine (*Muscle Nerve* 31: 520–527, 2005)

- a systematic review of the published scientific literature (1999 – 2003) and assesses the usefulness of EDX techniques in the diagnosis and prognostication of patients with peroneal neuropathy (499 articles identified)

- **complete recovery** (defined as the absence of weakness in the peroneal longus, AT, and EDB muscles, and by normal sensory findings): **100% had normal sensory conduction** velocities ***distal to the FH*** at the time of the initial study, **83% had normal motor conduction distal** to the FH along with slowing of motor conduction velocities ***across the FH***. Some subjects showed conduction block during the initial study, which had resolved at follow-up.
- **incomplete recovery** :had **abnormal motor NCSs distal** to the site as defined by either a decrease in amplitude (25%), absent response(37%), or mild slowing distally (25%). No subject with an absent response to the EDB with proximal stimulation had a full clinical recovery.
- On **electromyographic** testing, fibrillation potentials and positive sharp waves were more often present in those with incomplete recovery (86% vs. 50%)

Respiratory Failure and Hypoventilation Secondary to Neurosarcoidosis (Seattle, Washington)

- Electrodiagnostic findings consistent with bilateral phrenic neuropathy with **axon loss**, suggesting a poor prognosis.
- The **time course of recovery** was consistent with axon growth and reinnervation. An approximate distance from the mass to the diaphragm as measured on the chest CT was 10 to 15 cm. Assuming axonal growth of 1 mm/d, it would require 3 to 5 mo for reinnervation to occur. This study demonstrated evidence of reinnervation at 3 mo.
- The diaphragmatic electromyogram was particularly useful in quantifying the severity of the lesion, thus aiding in prognostication as well as clinical decision making.

Iranian Congress OF Electrodiagnosis
IN
Peripheral Nerve Lesions
DEC.21.22/2006
Tehran-Iran

Advances in Prognostication in Disorders of Consciousness *(H.I.)*

- Prognosis for what?
  - Prognosis for regaining consciousness
  - Prognosis for achieving a gross level of functional outcome
  - Prognosis for specific outcomes (e.g., ambulation, return to work)

- Variables of prognostic importance :
- • GCS
- • Time to follow commands
- • Age
- • Etiology
- • **ERPs**
- • Rate of functional change
- • Intracranial hematoma
- • Pupillary response

- MEPs/SSEPs/Event-related potentials:
- • SSEPs done within the first week, if bilaterally absent, suggest a poor prognosis. If present…???
- • Cognitive event related potentials may help distinguish good vs. poor prognosis among those with intact SSEPs
- • SSEPs done late (on conscious patients) are also predictive but behavioral measures are more useful
