# Supplementary material for: Meeting report on the first Iranian congress of electrodiagnosis in peripheral nerve lesions
Source: J Brachial Plex Peripher Nerve Inj. 2007 Apr 14;2:10. doi: 10.1186/1749-7221-2-10 (PMC1865540; doi:10.1186/1749-7221-2-10)
Supplement: Additional file 1 — Slides from the invited lectures and panel discussions. Compressed PDFs of 15 presentations and 2 panel discussions during the conference. [file 1749-7221-2-10-S1.zip › FACIAL PALSY.pdf]

Iranian Congress OF Electrodiagnosis
IN
Peripheral Nerve Lesions
DEC.21.22/2006
Tehran-Iran

Electrodiagnosis
in
Facial N. Lesion

M.H Bahrami M.D , physiatrist
Associate Professor Of PM&R
Shaheed Beheshti Medical University

Iranian Congress OF Electrodiagnosis
IN
Peripheral Nerve Lesions
DEC.21.22/2006
Tehran-Iran

Facial Nerve

VII Cranial N.

- Sensory : Tongue & Taste

- Motor : Facial Expression

- Autonomus

Iranian Congress OF Electrodiagnosis
IN
Peripheral Nerve Lesions
DEC.21.22/2006
Tehran-Iran

Etiology of Facial N. disorders

- **Bell`s palsy (idiopathic)**
- **Trauma & Tumors**
- **Viral (Herpes)**
- **Infection**
- **Birth trauma**
- **C.N.S**
- **Ear problem**
- **Polyneuritis**

Bell`s Palsy

- Idiopathic
  (viral , Autoimmune , Inflammatory)
- Sudden onset weakness
- Unilateral
- Associated complaints
- Good Recovery (85% in 3 wks)

Iranian Congress OF Electrodiagnosis
IN
Peripheral Nerve Lesions
DEC.21.22/2006
Tehran-Iran

Electrodiagnostic Evaluation

- Extremely valuable
- Diagnosis , Severity , prognosis
- Evaluated distal part
- Facial N. CMAP (latency & Amplitude)
- Side to side CMAP Amplitude
- Nerve Excitability Test (NET)
- Maximal Stimulation Test (MST)
- Blink Reflex
- Needle EMG
- Mag. Stimulation & F- wave

Iranian Congress OF Electrodiagnosis
IN
Peripheral Nerve Lesions
DEC.21.22/2006
Tehran-Iran

Facial CMAP

(ENOG , ENG , EEMG)

- Latency (< 4.2 ms)
- Amplitude & Side to side comparison
- Nasalis Muscle
- Supra maximal stimulation
- Severity & Prognosis
- Volume conduction
- Other Pitt Falls

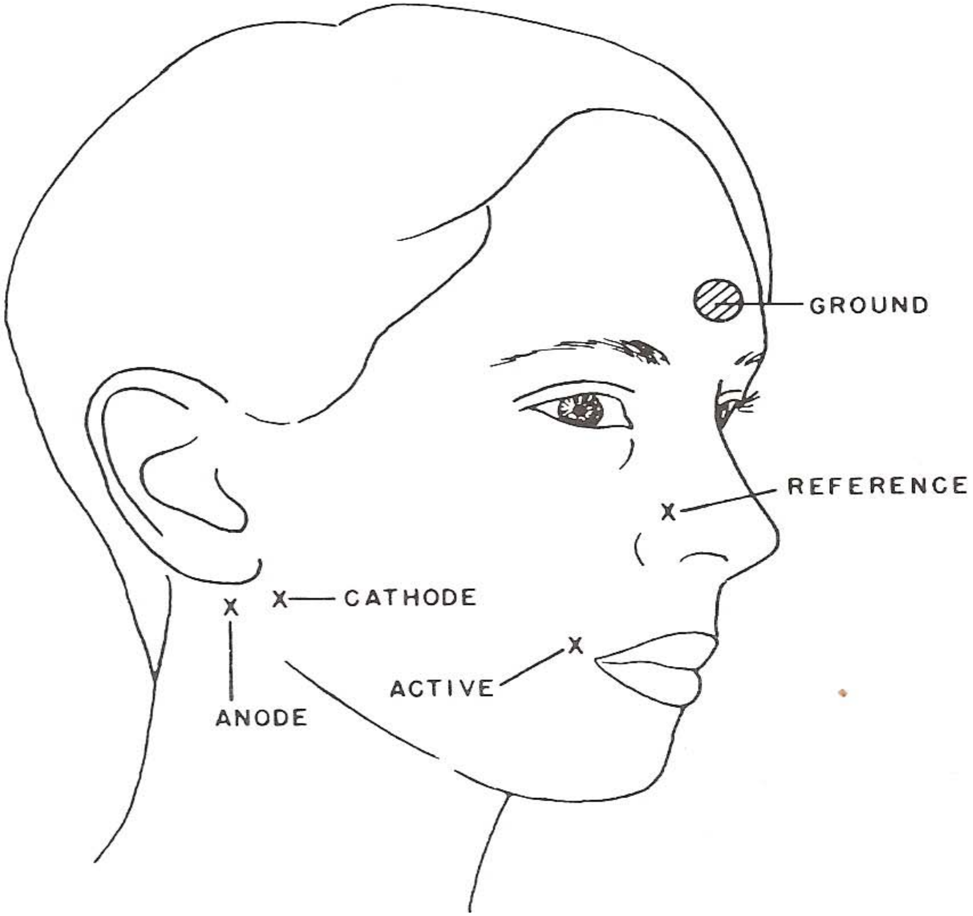

Figure 5 Facial nerve distal motor latency.

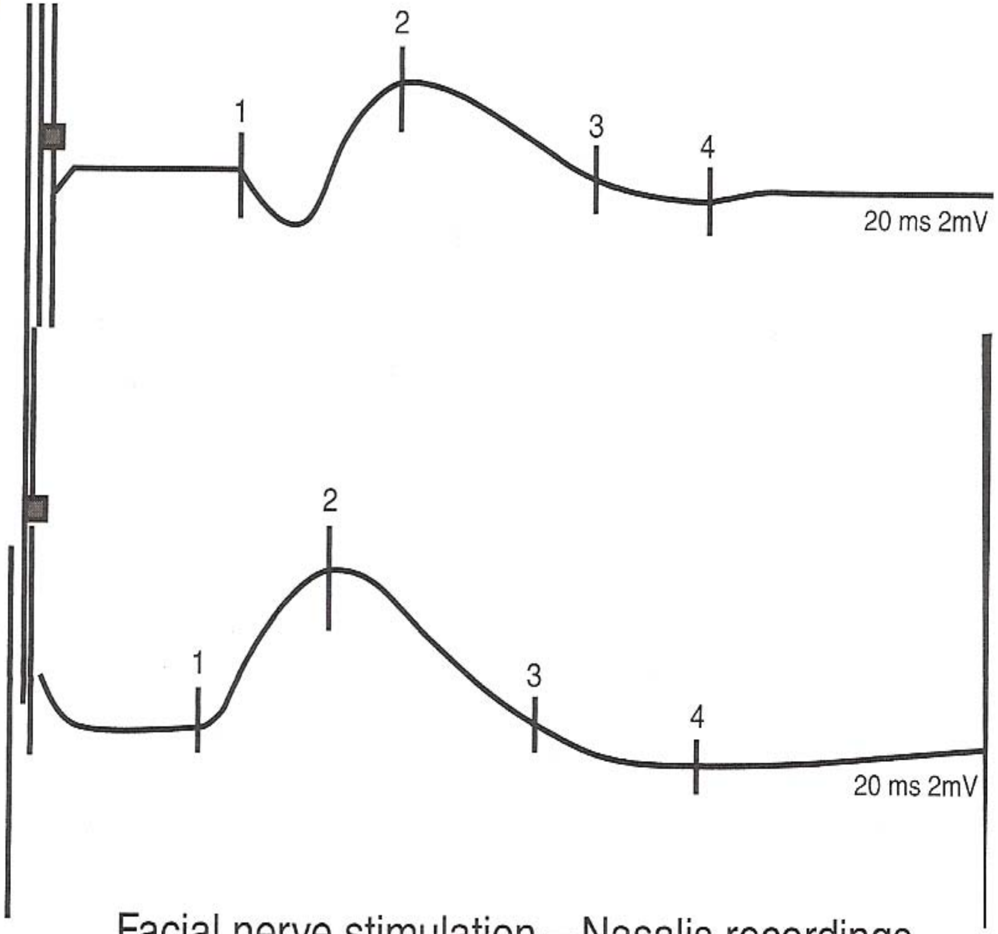

Facial nerve stimulation – Nasalis recordings

Figure 2-4. *CMAPs recorded from right and left nasalis muscles in facial nerve conduction studies. An initial positive deflection of CMAP in facial nerve conduction is common.*

Iranian Congress OF Electrodiagnosis
IN
Peripheral Nerve Lesions
DEC.21.22/2006
Tehran-Iran

Blink Reflex

- Corneal Reflex
- Fifth & seventh cranial N.
- Proximal & intracranial pathways
- R1 , R2 waves
- R/D Ratio
- Questionable prognostic value
- Synkinesis (in Lower Branches)
- Good localization of C.N.S damage

Iranian Congress OF Electrodiagnosis
IN
Peripheral Nerve Lesions
DEC.21.22/2006
Tehran-Iran

Blink Reflex & prognosis

Regain R1 in 4th week :

good outcome

Absent R1 & Direct Response :

poor prognosis

Absent R1 & good CMAP :

good prognosis

Iranian Congress OF Electrodiagnosis
IN
Peripheral Nerve Lesions
DEC.21.22/2006
Tehran-Iran

Needle EMG

- Diagnosis & Prognosis
- Denervation pot & MUAP changes
- Presence or absence of voluntary MUAP
- Evaluation of Regeneration
- Evaluation of synkinesis
- Long standing Evaluation

Prognosis

Bell`s Palsy after two weeks :

- **Axonal loss > 90% :**
  prolonged & incomplete recovery
- **Axonal loss < 90% :**
  90% satisfactory Recovery

5th day after injury is most reliable prognostic significance

(2-3 days delay & dynamic process)

Traumatic : Axonal loss > 90% :
(in 6th days) need surgery

Ramsy hunt syndrome: as Bell`s palsy

Iranian Congress OF Electrodiagnosis
IN
Peripheral Nerve Lesions
DEC.21.22/2006
Tehran-Iran

Prognostic Value of Tests

Test	Prognosis V/S Clinical
NET	Limited
CMAP onset latency	Limited
Needle EMG	Limited
CMAP (Side to Side)	Good
Blink Reflex	Uncertain
MST	Good

Iranian Congress OF Electrodiagnosis
IN
Peripheral Nerve Lesions
DEC.21.22/2006
Tehran-Iran

Electrodiagnosis

- Medical Consultation
- Diagnosis & Prognosis
- Decision Making
- Proper Treatment
