# Supplementary material for: Meeting report on the first Iranian congress of electrodiagnosis in peripheral nerve lesions
Source: J Brachial Plex Peripher Nerve Inj. 2007 Apr 14;2:10. doi: 10.1186/1749-7221-2-10 (PMC1865540; doi:10.1186/1749-7221-2-10)
Supplement: Additional file 1 — Slides from the invited lectures and panel discussions. Compressed PDFs of 15 presentations and 2 panel discussions during the conference. [file 1749-7221-2-10-S1.zip › MEDICAL MANAGEMENT OF NERVE LESIONS.pdf]

Iranian Congress OF Electrodiagnosis
IN
Peripheral Nerve Lesions
DEC.21.22/2006
Tehran-Iran

MEDICAL THERAPY IN PERIPHERAL NERVE INJURIES

1. REDUCING PAIN AND PARESTHESIA

- ANTIEPILEPTIC
- OPIOIDS
- ANTIDEPRESSANTS
- ENDOTHELIN RECEPTOR ANTAGONISTS
- CANABINOIDS
- MODULATORS OF ION CHANNELS
- LOCAL ANAESTHETIC AGENTS
- OTHERS; Lithium, Piracetam, Integrin, Vitamins…

Iranian Congress OF Electrodiagnosis
IN
Peripheral Nerve Lesions
DEC.21.22/2006
Tehran-Iran

2. NEUROPROTECTIVE AND REGENERATIVE

- NEUROTROPHICS
- CITICOLIN(Bcl-2 expression)
- LITHIUM(Bcl-2 expression)
- HEK-293 cells (release nerve growth factor in vivo)
- Aurintricarboxylic Acid , Sialidase ,Melatonin , Erythropoietin , Neuroprotective protein (ADNP) , Nitric Oxide ,

REDUCING PAIN AND PARESTHESIA

1. Antiepileptic drugs:

- Commonly used in nonepileptic situations
- Ectopic sites are active in nerve injury, redistribution and altered subunit composition (Ion channels)
- Improvement of acute pain management
- Combination with antidepressants
- Important central effect (the study with using muscimol and isoguvacine - zolpidem)

REDUCING PAIN AND PARESTHESIA

- Carbamazepin,
- Oxcarbamazepin,
- Lamotrigin,
- Gabapentine,
- Phenythoin,

REDUCING PAIN AND PARESTHESIA

- Antiepileptics ; mode of action :

  1-Potentiation of GABA transmission

  2-Reduction of glutamate mediated excitatory transmission

  3-Block of Voltage-activated ion channels

REDUCING PAIN AND PARESTHESIA

- Unmyelinated C-fiber nerve axons
  DM is an example

- DRG role

Iranian Congress OF Electrodiagnosis
IN
Peripheral Nerve Lesions
DEC.21.22/2006
Tehran-Iran

REDUCING PAIN AND PARESTHESIA

2-Opioids :

- All are agonist of mu-receptor
- d-1 isomer of Methadone was the strongest against neuropathic pain in one study
- Seems they could be considered in such application

REDUCING PAIN AND PARESTHESIA

3-Antidepressants :

An study using Venlafaxin and GABA pentine showed reducing effect (inc. urinary excretion)

Another study with Imipramin and GABApentine indictes time table mode of action for imipramin (4th week) but GABA pentine has dose dependent type of action

REDUCING PAIN AND PARESTHESIA

4- Canabinoids :

They are promising drugs ,now in experimental diabetic neuropathic pain,

Dose dependent antinociceptive effect

REDUCING PAIN AND PARESTHESIA

5- Drugs affecting Ion channels:

Drugs that alter channels activity may have benefit in N.I.

In N.I. (and normal nerves also), Fluorescein accumulates in DRG after different ways of injection

A major consequence of the remodeling is increased excitability

REDUCING PAIN AND PARESTHESIA

A-Potassium channel:

4-aminodpyridine subtype

B-Sodium channel:

Subtypes of tetrodotoxin resistant voltage-gated sodium channels are involved

TCA drugs,anticonvulsants,Lidocaine ,mexiletine have affects on it

REDUCING PAIN AND PARESTHESIA

C-Calcium channel:

-May be important in chemotherapy induced pain ;Vincristin , Paclitaxel

-In a study all four Ca++ reducing drugs inhibited allodynia

-At the spinal level blocking of several of them notably N type can prominently alter pain behavior

REDUCING PAIN AND PARESTHESIA

- E- Glutamate receptor:

This receptor's activity has an important role in the level of peripheral nerve , spinal cord and supraspinal levels

MK-801 can reduce pain and also it has some effects on NMDA receptor (now researches on this receptor shows no effect)

Iranian Congress OF Electrodiagnosis
IN
Peripheral Nerve Lesions
DEC.21.22/2006
Tehran-Iran

REDUCING PAIN AND PARESTHESIA

- D- GABA receptor agonists:

Selective GABA receptor agonists,

Gaboxadol dose dependently reduced allodynia and hyperalgesia

Muscimol and Isoguavacine not affected pains

(Central and Peripheral acting difference)

REDUCING PAIN AND PARESTHESIA

- 6- Endothelin receptor antagonists

Endothelin can contribute to nociceptive changes in animal model of inflammatory ,cancer and diabetic induced pain

Bosentin,Atrasentin

REDUCING PAIN AND PARESTHESIA

- 7- Thiamine,Pyridoxin,Cyanocobalamin :

Produced inhibition of thermal hyperalgesia

not affected mechanical hyperalgesia

Iranian Congress OF Electrodiagnosis
IN
Peripheral Nerve Lesions
DEC.21.22/2006
Tehran-Iran

REDUCING PAIN AND PARESTHESIA

- 8- Local anesthetic agents

They have a possible role in nerves blood flow

Levobupivacaine and Ropivacaine has been studied in a rat model with sciatic nerve injury

showed significant blood reduction and less histopathological damages

(the reason ,unknown)

Iranian Congress OF Electrodiagnosis
IN
Peripheral Nerve Lesions
DEC.21.22/2006
Tehran-Iran

REDUCING PAIN AND PARESTHESIA

- 9-Others :

Piracetam and Lithium:

Some evidences show their efficacy in the case of pain and paresthesia

NEUROPROTECTIVE AND REGENERATIVE

Tissue engineering strategies :

Hek – 209 cells ; they can release nerve growth factor (NGF) in vitro also tried in vivo and was effective

Iranian Congress OF Electrodiagnosis
IN
Peripheral Nerve Lesions
DEC.21.22/2006
Tehran-Iran

NEUROPROTECTIVE AND REGENERATIVE

Neurotrophic agents

Brain derived neurotrophic factor (BDNF)

Glial derived neurotrophic factor (GDNF)

Topicl application of them in 30 min. significantly improved motor function and reduced BSCB breakdown and edema

NEUROPROTECTIVE AND REGENERATIVE

Activation of neuroprotective pathways by metabotropic group 1 glutamate receptors

Neuroprotection mediated via mGluR 1

A study with cell death induced by NMDA activation

NEUROPROTECTIVE AND REGENERATIVE

Others :

Citicoline and Lithium:

Improving retinal ganglion cell survival and axon regeneration

The effect is via an increase in Bcl-2 expression

Maybe preventing of cell death

Iranian Congress OF Electrodiagnosis
IN
Peripheral Nerve Lesions
DEC.21.22/2006
Tehran-Iran

NEUROPROTECTIVE AND REGENERATIVE

Some chemical agents have been used for this issue

Aurintricarboxylic Acid:

improved regeneration in optic nerve cut axons
