# Supplementary material for: Meeting report on the first Iranian congress of electrodiagnosis in peripheral nerve lesions
Source: J Brachial Plex Peripher Nerve Inj. 2007 Apr 14;2:10. doi: 10.1186/1749-7221-2-10 (PMC1865540; doi:10.1186/1749-7221-2-10)
Supplement: Additional file 1 — Slides from the invited lectures and panel discussions. Compressed PDFs of 15 presentations and 2 panel discussions during the conference. [file 1749-7221-2-10-S1.zip › NERVE LESION CLASSIFICATION.pdf]

Iranian Congress OF Electrodiagnosis
IN
Peripheral Nerve Lesions
DEC.21.22/2006
Tehran-Iran

Acute Peripheral Nerve Injury

Dr. Ali Reza Zalie
Associate Professor of Neurosurgery
SBMU

Iranian Congress OF Electrodiagnosis
IN
Peripheral Nerve Lesions
DEC.21.22/2006
Tehran-Iran

Approach to the Patient

- The approach to the patient with a peripheral nerve problem is fundamentally clinical.

- The neurological examination remains the cornerstone in evaluating a peripheral nerve condition and has not been easily supplanted by imaging advances.

- The majority of peripheral nerve problems either improve or remain unchanged, whereas only a small minority of conditions worsen.

Iranian Congress OF Electrodiagnosis
IN
Peripheral Nerve Lesions
DEC.21.22/2006
Tehran-Iran

ER Management

- The ER management of a patient who is suspected of having sustained peripheral nerve injury differs greatly from that of the patient who is seen electively or even urgently in the clinical setting.

- In any trauma patient, attention to life-threatening airway, respiratory, circulatory, and CNS injuries always take first priority before limb injuries are addressed.

Iranian Congress OF Electrodiagnosis
IN
Peripheral Nerve Lesions
DEC.21.22/2006
Tehran-Iran

ER Evaluation

- In multitrauma victims, nerve injuries and brachial plexus injuries are relatively frequent, affecting 5% and 1% of trauma patients, respectively.
- These injuries can be diagnosed at the initial trauma encounter in more than 60% of cases.
- In patients with an altered level of consciousness, an asymmetrical neurological examination, with loss of function confined to one limb when accompanied by loss of DTRs, can be suggestive of nerve injury.

Iranian Congress OF Electrodiagnosis
IN
Peripheral Nerve Lesions
DEC.21.22/2006
Tehran-Iran

ER Evaluation

- The precise distribution of nerve injury, even in the comatose patient, can also be ascertained by assessing the lack of autonomic function (sweating and loss of wrinkling after immersion of hand or foot in water), as suggested by Kline and Hudson's.

- Any patient with a soft tissue, tendon, bone, joint, or vascular injury in the limb should be examined for nerve damage.

- The rule of thumb to exclude a nerve injury is to verify that the most distal aspect of the nerve is functioning.

Iranian Congress OF Electrodiagnosis
IN
Peripheral Nerve Lesions
DEC.21.22/2006
Tehran-Iran

Clinical Dx

- Preservation of *any* function (autonomic, sensory to autonomous distributions, and especially motor) in the distribution of the nerve or nerve element is noted.

- Such partial injuries are by their nature less likely to be severe and often exhibit spontaneous recovery over time.

- Conversely, complete injuries may or may not recover.

Open Injuries

- In sharp penetrating trauma (e.g., glass, knife, and razor blade injuries) with nerve injury, primary exploration and suture repair of the divided nerve, aided by micro-techniques and magnification, in the operating room is best.
- At times, the nerve is found to be merely contused or bruised during exploration, as in the majority of patients with nerve damage from gunshot wounds or high-velocity missiles.
- The contused and divided nerve ends are sutured, using large non-absorbable suture, to fascial tissue under some distraction (to minimize retraction) adjacent to each other.
- The definitive nerve repair is performed at a secondary exploration after several weeks have elapsed to allow the extent of longitudinal injury to declare itself."

Iranian Congress OF Electrodiagnosis
IN
Peripheral Nerve Lesions
DEC.21.22/2006
Tehran-Iran

NERVE INJURY GRADING AND TIMING OF SURGERY

- One important goal of the history and physical examination is to gain an appreciation of the severity of injury to the affected nerve element.

- Seddon's elegant three-grade (neuropraxia, axonotrnesis, and neurotrnesis) classification scheme, based on clinicopathologic correlation, remains useful.

- It benefits from its brevity and relevance in predicting natural history and outcome.

- The modification of the Seddon scale to the five-point grading system by Sunderland provides a somewhat more accurate anatomic appreciation of the damage to the internal nerve structure

Iranian Congress OF Electrodiagnosis
IN
Peripheral Nerve Lesions
DEC.21.22/2006
Tehran-Iran

Neuropraxia

- In *neuropraxia* (Sunderland grade 1), the mildest form of injury, there is a reversible conduction block, manifested clinically as loss of function, which persists for hours to days.“
- There is either minimal or no discernible histopathologic alteration in nerve structure.
- In some (more severe) neuropraxic injuries, axons have localized thinning and mild segmental demyelination.

Neuropraxia

- The initial clinical examination often shows incomplete and sparing of autonomic function.
- In patients exhibiting complete loss, an initial single clinical assessment cannot distinguish neuropraxia from more severe injuries.
- Grade 1 injuries are characterized by excellent spontaneous recovery over days to weeks and rarely over some months.

Iranian Congress OF Electrodiagnosis
IN
Peripheral Nerve Lesions
DEC.21.22/2006
Tehran-Iran

Axonotmesis

- In an *axonotmetic* (Sunderland grade 2) lesion, axon continuity is disrupted but with relative sparing of the connective tissue structure of the nerve, the fascicular integrity is maintained, as is the fine endoneurial network, with minimal endoneurial edema and fibrosis.

- After division of the nerve fiber, wallerian degeneration occurs in the distal axon. Degeneration of the axon also occurs for a variable distance proximal to the site of nerve injury.

- The elongating tips of regenerating axons are guided toward the end-organ by the intact endoneurial basement membrane.

- The rate of regeneration averages approximately 1 mm a day or an inch per month, parameters that are useful in serial clinical evaluation of the patient while awaiting possible return of function.

Iranian Congress OF Electrodiagnosis
IN
Peripheral Nerve Lesions
DEC.21.22/2006
Tehran-Iran

Axonotmesis

- Grade 2 injuries often recover effectively without the need for operative intervention.

- The completeness of recovery is governed by factors such as the location of the injury and the specific nerve or nerve element involved.

- The rate of regeneration averages approximately 1 mm a day or an inch per month, useful in serial clinical evaluation while awaiting possible return of function.

Iranian Congress OF Electrodiagnosis
IN
Peripheral Nerve Lesions
DEC.21.22/2006
Tehran-Iran

Higher Grade Lesions

- When the damage is confined to the membranous structures within the fascicle, a Sunderland grade 3 lesion is present

- Additional involvement of extrafascicular connective tissue denotes a grade 4 injury with variable degree of intrafascicular fibrosis resulting in, frustration of regenerating axons and leading to their aberrant regrowth, despite gross continuity of the nerve itself.

- The resulting *neuroma in continuity* contains a meshwork of connective tissue entwined with fine-caliber, poorly myelinated axons.

Neurotmesis

- In a grade 5 injury, there is anatomic severance *(neurotmesis)* of the entire nerve.
- Grade 5 injuries almost always require repair, and the only important consideration is the timing of repair.
- For sharply divided nerves (e.g., laceration by glass or knife), acute repair within hours to a day or two is ideal.
- More bluntly lacerated nerves are repaired 3 to 4 weeks after injury. This delay allows the longitudinal extent of injury to be fully delineated and declared so that debridement of the nerve to healthy proximal and distal stumps can be performed before repair.

Iranian Congress OF Electrodiagnosis
IN
Peripheral Nerve Lesions
DEC.21.22/2006
Tehran-Iran

Management Protocol

- Return of function over time depends to a great extent on the underlying neuropathologic condition of the nerve; those with a large neurotrnetic component do not generally recover, whereas those with a neuropraxic or axonotmetic pathology, or both, may recover."

- The primary indication for operative exploration of the nerve injury in continuity is lack of clinical or electrophysiologic recovery.

- In practice, more proximal nerve injuries and distal injuries not exhibiting spontaneous recovery are candidates for exploration,

Iranian Congress OF Electrodiagnosis
IN
Peripheral Nerve Lesions
DEC.21.22/2006
Tehran-Iran

Time of Exploration

- The optimal timing for exploration of a nerve injury that is in continuity is greatly influenced by the mechanism of injury.

- Injuries that are relatively more focal such as those produced by gunshot wounds, iatrogenic causes, stab wounds, lacerations, and fracture-associated contusions are best explored 2 to 3 months after wounding.

- Lengthier lesions resulting from severe contusion or stretch are ideally explored 4 to 5 months after onset.

Iranian Congress OF Electrodiagnosis
IN
Peripheral Nerve Lesions
DEC.21.22/2006
Tehran-Iran

Prognosis

- Clinical outcome differs from no return to a very good return of function in grade 3 injuries.
- In contrast, the grade 4 injury represents the most severe pathology for a neuroma in continuity.
- Clinical recovery seldom occurs, unless operative resection and repair are undertaken.

Iranian Congress OF Electrodiagnosis
IN
Peripheral Nerve Lesions
DEC.21.22/2006
Tehran-Iran

MECHANISMS OF INJURY

- Traction, Stretch, and Contusion
- Laceration
- Missiles (Gunshot Wounds)
- Compression and Ischemia
- Thermal and Electrical Injuries
- Injection Injury
- Iatrogenic Injuries

Iranian Congress OF Electrodiagnosis
IN
Peripheral Nerve Lesions
DEC.21.22/2006
Tehran-Iran

Injection Injuries

- Injury to peripheral nerves secondary to injection is a serious complication of intramuscular drug administration.
- Any nerve is at risk, but the proximal radial nerve and the sciatic nerve in the buttock are by far the most common ones injected.
- Damage may occur from the needle itself, but mostly it is secondary to the toxic effects of the drug or agent being instilled in the intra-neural compartment.

Iranian Congress OF Electrodiagnosis
IN
Peripheral Nerve Lesions
DEC.21.22/2006
Tehran-Iran

Injection Injuries

- In the typical scenario, needle placement results in an immediate electric-like shock sensation down the extremity. Concomitantly, on injection of the agent, severe radiating pain and paresthesias result.

- The patient usually experiences a severe pain described with adjectives such as burning, searing, electrical-or a numbing sensation along the course of the injected nerve occurs.

- In approximately 10% of cases, a delayed onset of neuropathy occurs the symptoms are often less dramatic, described variously as a burning pain, a deep discomfort, or bothersome paresthesias down the limb and in the distribution of the affected nerve.

- When incomplete, motor loss is usually greater than sensory neuritic pain, of variable intensity and often accompanies the neurological deficit.

Iranian Congress OF Electrodiagnosis
IN
Peripheral Nerve Lesions
DEC.21.22/2006
Tehran-Iran

Management Guidelines

- Management of the patient with a peripheral nerve injection injury essentially follows the guidelines established for any patient with a nerve lesion in continuity.

- Most partial injuries and some complete injuries recover without operative intervention, with early return of function appearing to be the most significant prognostic factor in these cases.“

- Patients not exhibiting spontaneous recovery over approximately 4 months, as well as the occasional patient with medically intractable neuritic pain syndrome, are candidates for surgical exploration of the injury site, with external and internal neurolysis and nerve repair, depending on intraoperative findings.
