# Supplementary material for: Meeting report on the first Iranian congress of electrodiagnosis in peripheral nerve lesions
Source: J Brachial Plex Peripher Nerve Inj. 2007 Apr 14;2:10. doi: 10.1186/1749-7221-2-10 (PMC1865540; doi:10.1186/1749-7221-2-10)
Supplement: Additional file 1 — Slides from the invited lectures and panel discussions. Compressed PDFs of 15 presentations and 2 panel discussions during the conference. [file 1749-7221-2-10-S1.zip › NON-ROUTIN EDX.pdf]

Iranian Congress OF Electrodiagnosis
IN
Peripheral Nerve Lesions
DEC.21.22/2006
Tehran-Iran

به نام خدا

Non Routine Electrodiagnosis

Iranian Congress OF Electrodiagnosis
IN
Peripheral Nerve Lesions
DEC.21.22/2006
Tehran-Iran

Collision Technique

Response is obtained by using two stimuli
Same or different nerves
Collision of antidromic and orthodromic impulses

Two principle:

1. **nerve excitation travel orthodromically and antidromically**
2. **Proximal and distal potentials cancel each other between two points of stimulation**

Collision Technique-

1. To record F-Wave with stimulation at the proximal site of nerves

2. To isolate the CMAP of ulnar or median at the axilla in CTS or ulnar neuropathy at the elbow

3. To isolate the median CMAP from the ulnar response through the Martin-Grober anastomosis

Iranian Congress OF Electrodiagnosis
IN
Peripheral Nerve Lesions
DEC.21.22/2006
Tehran-Iran

Refractory period

Period of inexcitability after the actionpotential
Two phase : absolute relative

By paired stimulation with varying intervals

Sensory or mixed nerve conduction

More sensitive than conventional NCS in detection of neuropathy

Iranian Congress OF Electrodiagnosis
IN
Peripheral Nerve Lesions
DEC.21.22/2006
Tehran-Iran

Intraoperative Nerve Conduction

Complete nerve lesion
No clinical or electrophysiological improvement within 2-4 month
Surgical exploration

Surgical exploration → Completely severed → Must be sutured

→ Lesion in continuity → Intraoperative CNAP

Iranian Congress OF Electrodiagnosis
IN
Peripheral Nerve Lesions
DEC.21.22/2006
Tehran-Iran

Intraoperative CNAP instrumentation

Modern EMG machines

**Filter setting**	→	**20 Hz to 2 KHz**
**sensitivity**	→	**50-100 microvolt**
**Sweep speed**	→	**0.5-1 ms**
**Stimulation intensity**	→	**1-10 mA 20**

Gas sterilization: Electrodes
Holder
Cables

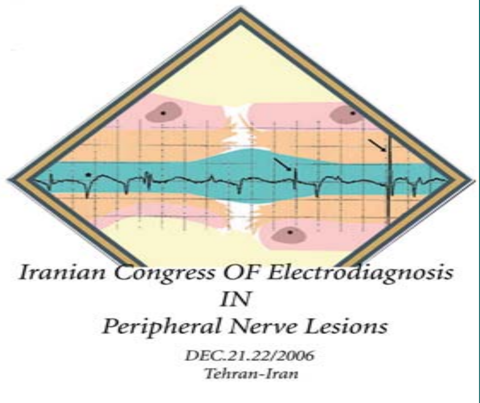

Intraoperative CNAP

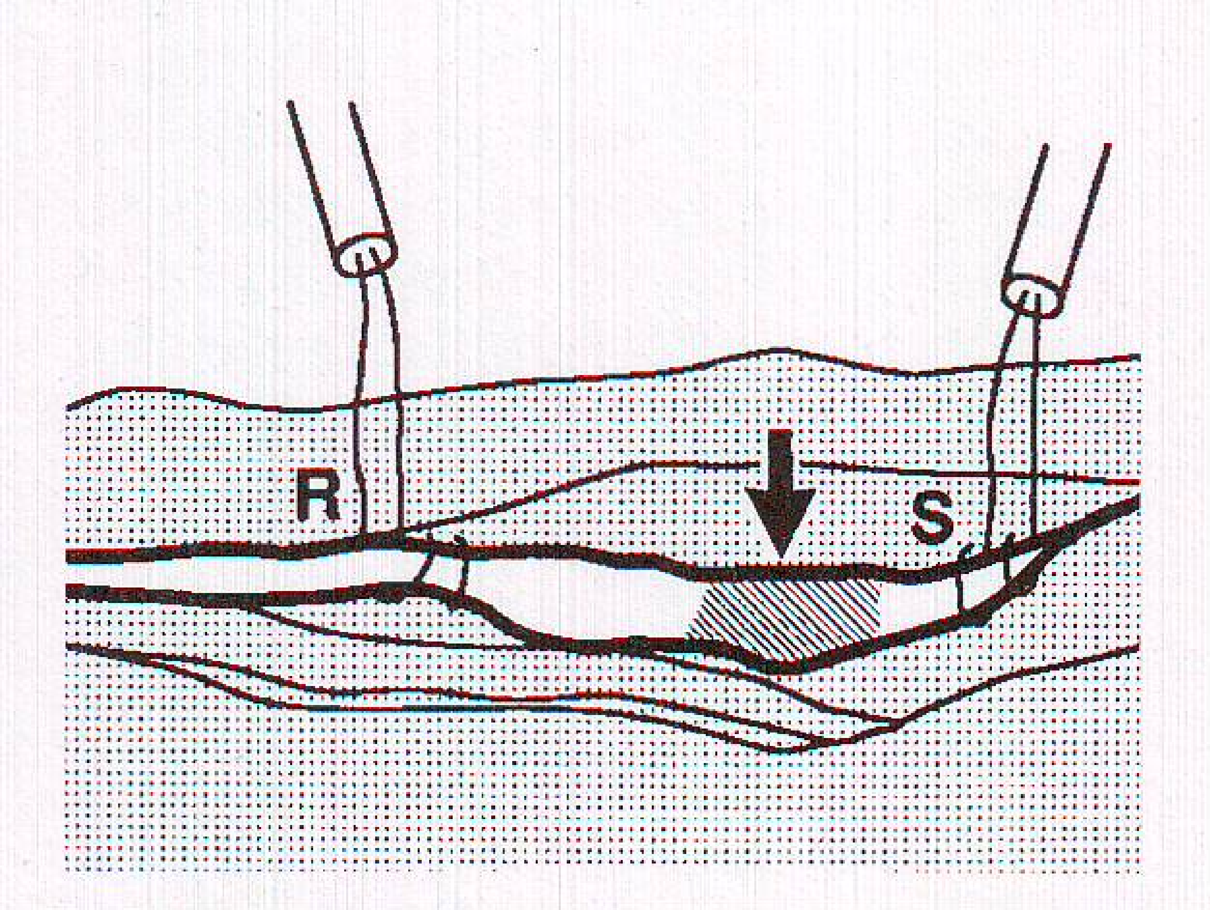

Important Surgical Factors

The tourniquet 15-20 min

Neuromuscular Agents

Exposure of the nerve 5-6 cm

Iranian Congress OF Electrodiagnosis
IN
Peripheral Nerve Lesions
DEC.21.22/2006
Tehran-Iran

Intraoperative CNAP results

Presence

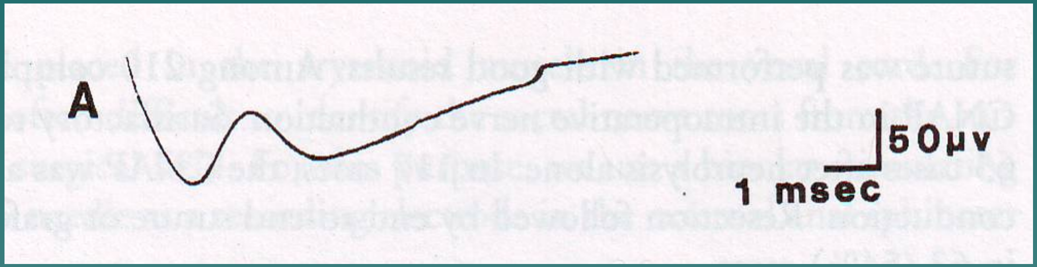

neurolysis

Absence

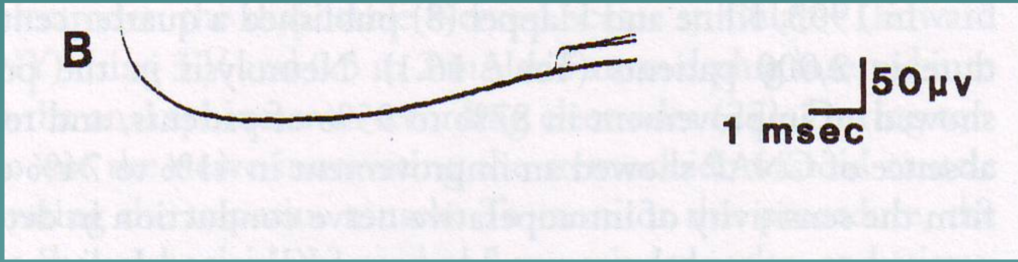

Neuroraphy or nerve graft

Iranian Congress OF Electrodiagnosis
IN
Peripheral Nerve Lesions
DEC.21.22/2006
Tehran-Iran

Inching Technique
short segmental stimulation

Ordinary NCS → **Distance 10 cm**
Approximate site of compression

Inching → **1-2 cm**
Precise of localization

Focal neuropathies :

Ulnar-Elbow

Peroneal –Head of fibula

Median – Carpal tunnel

Iranian Congress OF Electrodiagnosis
IN
Peripheral Nerve Lesions
DEC.21.22/2006
Tehran-Iran

actical impotence of inching tech

Ulnar nerve ---different therapeutic approach

- Lesion is localized to more than 2 cm distal to the medial epicondyl

Cubital tunnel syndrome → **Decompression**

- Lesion is localized to the medial epicondyl or proximal to it

Epicondylar Compression –Tardy ulnar palsy → **Anterior transposition**

Iranian Congress OF Electrodiagnosis
IN
Peripheral Nerve Lesions
DEC.21.22/2006
Tehran-Iran

Motor Unit Number Estimation
MUNE

Principle: to compare some averaged values of single motor units With the value for the entire muscle and thereby to determine The number of motor units in that muscle

$$\text{MUNE} = \frac{\text{CMAP (amplitude or area)}}{\text{Average S.MUP}}$$

- **Maximal stimulation of any motor nerve activates all the muscle Innervated by the point of stimulation**
- **All motor axons are activated by maximal stimulation**
- **The size of an s-mup is constant**

Iranian Congress OF Electrodiagnosis
IN
Peripheral Nerve Lesions
DEC.21.22/2006
Tehran-Iran

MUNE- Mcmans manual increment method

Machine setup: motor nerve conduction

Recording: strip electrodes over the muscles

Stimulation: the stimulus intensity to a motor nerve is gradually raised above the threshold level and the response is observed to grow in steps. obtain ten or so increments
