# Supplementary material for: Meeting report on the first Iranian congress of electrodiagnosis in peripheral nerve lesions
Source: J Brachial Plex Peripher Nerve Inj. 2007 Apr 14;2:10. doi: 10.1186/1749-7221-2-10 (PMC1865540; doi:10.1186/1749-7221-2-10)
Supplement: Additional file 1 — Slides from the invited lectures and panel discussions. Compressed PDFs of 15 presentations and 2 panel discussions during the conference. [file 1749-7221-2-10-S1.zip › PHYSICAL MODALITIES IN NERVE LESIONS.pdf]

Iranian Congress OF Electrodiagnosis
IN
Peripheral Nerve Lesions
DEC.21.22/2006
Tehran-Iran

In the name of

GOD

Iranian Congress OF Electrodiagnosis
IN
Peripheral Nerve Lesions
DEC.21.22/2006
Tehran-Iran

Physical medicine modalities in peripheral nerve lesions

P bonakdar MD
Chamran hospital

MODALITIES

- *US*
- *IR*
- *SWD*
- *LASER*
- *ES*
- *MW*

Iranian Congress OF Electrodiagnosis
IN
Peripheral Nerve Lesions
DEC.21.22/2006
Tehran-Iran

PERIPHERAL NERVE LESION

- *ULNAR*
- *MEDIAN*
- *RADIAL*
- *BRACHIAL PLEXUS*
- *SCIATIC*
- *FEMORAL*
- *TIBIAL*
- *PERONEAL*

Iranian Congress OF Electrodiagnosis
IN
Peripheral Nerve Lesions
DEC.21.22/2006
Tehran-Iran

3 SYMOLIC MODELS

EVIDENCE BASED VIEW

- *SCIENTIFIC REVIEWS*
- *PATHOPHYSIOLOGIC VIEW*

Iranian Congress OF Electrodiagnosis
IN
Peripheral Nerve Lesions
DEC.21.22/2006
Tehran-Iran

1ST MODEL

- *ENTRAPMENTS*

- ***CTS***

Iranian Congress OF Electrodiagnosis
IN
Peripheral Nerve Lesions
DEC.21.22/2006
Tehran-Iran

2th MODEL

- *TRAUMATIC INJURY*

  WITH SURGICAL REPAIR

  WITHOUT SURGICAL REPAIR

Iranian Congress OF Electrodiagnosis
IN
Peripheral Nerve Lesions
DEC.21.22/2006
Tehran-Iran

3th MODEL

- *GENERALIZED*

  POLYNEUROPATHY

Iranian Congress OF Electrodiagnosis
IN
Peripheral Nerve Lesions
DEC.21.22/2006
Tehran-Iran

EVIDENCE BASED VIEW

Iranian Congress OF Electrodiagnosis
IN
Peripheral Nerve Lesions
DEC.21.22/2006
Tehran-Iran

Cochrane non surgical treatment of *cts*

- Injection and oral steroid
- Splinting
- Yoga
- Carpal bone mobilization
- us

Iranian Congress OF Electrodiagnosis
IN
Peripheral Nerve Lesions
DEC.21.22/2006
Tehran-Iran

Laser and cts

- *7 study*

- *5 +ive (10-30j / cm2)*
- *2 –ive (1.8-6 j /cm2)*

ELECTRICAL STIMULATION

Iranian Congress OF Electrodiagnosis
IN
Peripheral Nerve Lesions
DEC.21.22/2006
Tehran-Iran

E.S. in Braddom

1996 - 2007

Iranian Congress OF Electrodiagnosis
IN
Peripheral Nerve Lesions
DEC.21.22/2006
Tehran-Iran

torkestan

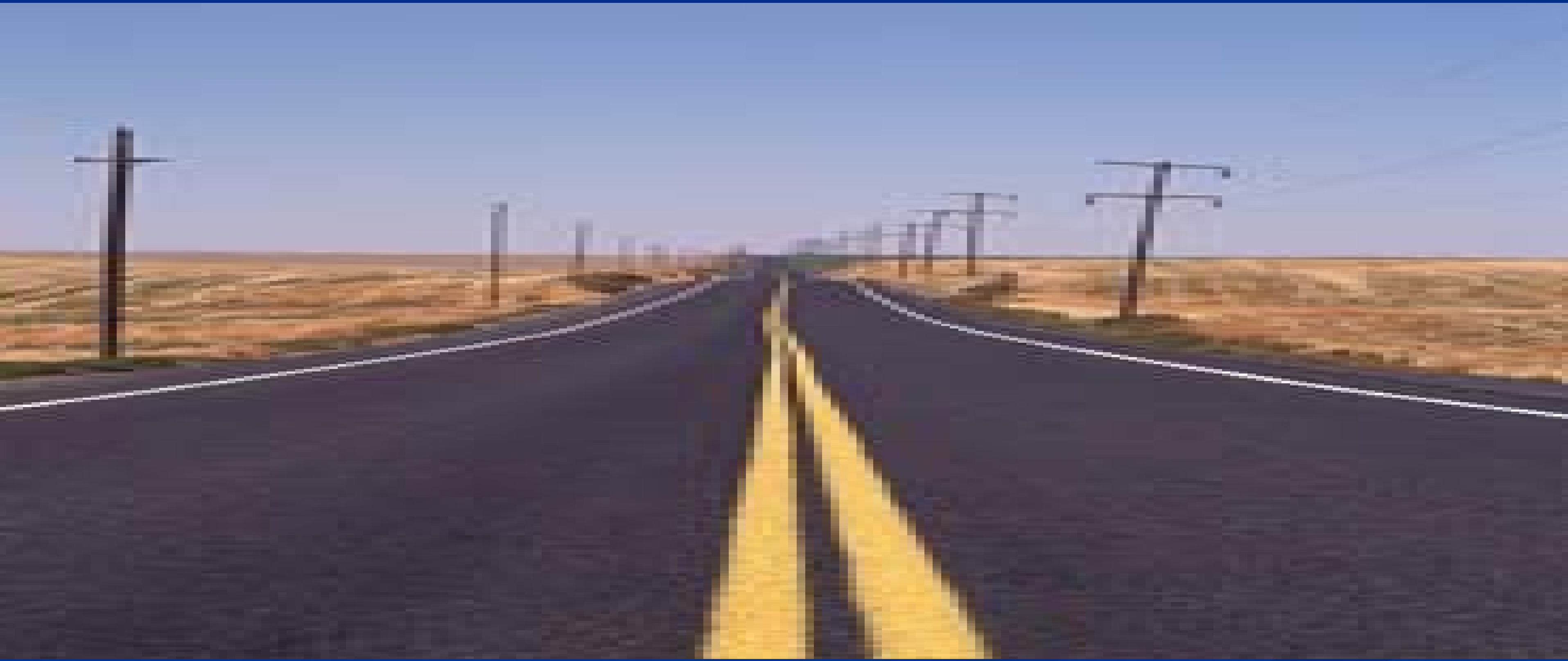

Iranian Congress OF Electrodiagnosis
IN
Peripheral Nerve Lesions
DEC.21.22/2006
Tehran-Iran

LASER

Laser : studies

- Axonotmesis model
- 1980s (crushed rat sciatic peroneal nerve)
- Short and long term
- Local and spinal
- Surgical repair model (postoperative)4 study

Iranian Congress OF Electrodiagnosis
IN
Peripheral Nerve Lesions
DEC.21.22/2006
Tehran-Iran

Laser : wavelength

- *HeNe 632.8 nm*
- *780-830 nm*
- *> 904 nm*

Iranian Congress OF Electrodiagnosis
IN
Peripheral Nerve Lesions
DEC.21.22/2006
Tehran-Iran

Laser : energy density

10 J/cm2

150 J/cm2

Iranian Congress OF Electrodiagnosis
IN
Peripheral Nerve Lesions
DEC.21.22/2006
Tehran-Iran

Laser : mechanism of action on nervous system

- *Increase of ATP synthesis*
- *Stimulate axonal sprouting*
- *Increase growth associated protein gap-34*
- *Reducing degeneration of spinal cord neuron*
- *Supress neurotoxic agents*
- *Stimulate proliferation of schwann cells*

Iranian Congress OF Electrodiagnosis
IN
Peripheral Nerve Lesions
DEC.21.22/2006
Tehran-Iran

Laser : type of emission

- Continuous
- pulsed

Laser : type and site of application

- *Transcutaneous*
- *direct*

Iranian Congress OF Electrodiagnosis
IN
Peripheral Nerve Lesions
DEC.21.22/2006
Tehran-Iran

laser

- Early
- late

ES

- > 20% axonal mass
- Good prognosis
- Low fr.
- Long dur.

Iranian Congress OF Electrodiagnosis
IN
Peripheral Nerve Lesions
DEC.21.22/2006
Tehran-Iran

ES

- ***Feedback stimulation***

Iranian Congress OF Electrodiagnosis
IN
Peripheral Nerve Lesions
DEC.21.22/2006
Tehran-Iran

THIS IS THE PROBLEM

- ***PROXIMAL***

OR

- ***DISTAL***

Iranian Congress OF Electrodiagnosis
IN
Peripheral Nerve Lesions
DEC.21.22/2006
Tehran-Iran
