# Supplementary material for: Meeting report on the first Iranian congress of electrodiagnosis in peripheral nerve lesions
Source: J Brachial Plex Peripher Nerve Inj. 2007 Apr 14;2:10. doi: 10.1186/1749-7221-2-10 (PMC1865540; doi:10.1186/1749-7221-2-10)
Supplement: Additional file 1 — Slides from the invited lectures and panel discussions. Compressed PDFs of 15 presentations and 2 panel discussions during the conference. [file 1749-7221-2-10-S1.zip › PROXIMAL MEDIAN NERVE LESION.pdf]

Iranian Congress OF Electrodiagnosis
IN
Peripheral Nerve Lesions
DEC.21.22/2006
Tehran-Iran

Median nerve neuropathies (proximal part)

Dr. M.T. Hollisaz

Associate professor of physical medicine and Rehabilitation

Baqiyatallah university

Iranian Congress OF Electrodiagnosis
IN
Peripheral Nerve Lesions
DEC.21.22/2006
Tehran-Iran

median nerve neuropathies

- Axillary median nerve lesion
- Arm region
- Distal arm proximal forearm region

Iranian Congress OF Electrodiagnosis
IN
Peripheral Nerve Lesions
DEC.21.22/2006
Tehran-Iran

Axillary median nerve lesion

- Misuse of crutches
- missile injuries
- Arteriography
- Needle puncture
- Hematoma
- Sleeping in an awkward position
- Therapeutic fistulae

Iranian Congress OF Electrodiagnosis
IN
Peripheral Nerve Lesions
DEC.21.22/2006
Tehran-Iran

Arm region

- ☞ Humeral fractures
- ☞ Laceration
- ☞ bullet injury
- ☞ Brachial fistula
- ☞ prolonged tourniquet
- ☞ Hanging over chair back

Iranian Congress OF Electrodiagnosis
IN
Peripheral Nerve Lesions
DEC.21.22/2006
Tehran-Iran

[illegible]ctrodiagnosis Evaluation

- Median SNAP
- CMAP
- NEEDLE

Iranian Congress OF Electrodiagnosis
IN
Peripheral Nerve Lesions
DEC.21.22/2006
Tehran-Iran

Distal arm

- Gun shot
- Fractures/ Dislocation
- Lacertus fibrosus
- Supra condylar spur
- Pronator teres
- Flexor digitorum superficialis fibrous ridge

Iranian Congress OF Electrodiagnosis
IN
Peripheral Nerve Lesions
DEC.21.22/2006
Tehran-Iran

Lacertus fibrosus (Bicipital aponeurosis)

- Clinical features (Elbow pain)
- EDX

Iranian Congress OF Electrodiagnosis
IN
Peripheral Nerve Lesions
DEC.21.22/2006
Tehran-Iran

Supracodylar spur
Ligament of struthers

- Clinical features
  - Sharp Pain
  - Tinnel Sign
  - Weakness
- EDX

Iranian Congress OF Electrodiagnosis
IN
Peripheral Nerve Lesions
DEC.21.22/2006
Tehran-Iran

Pronator teres syndrome
(sublimis bridge)

- Hyperotro phied prona tor teres
- Repeated pronationl supination
- Force ful pronation
- Anomalous arteries
- Post op. scar

Iranian Congress OF Electrodiagnosis
IN
Peripheral Nerve Lesions
DEC.21.22/2006
Tehran-Iran

Pronator teres syndrome

- Clinical : insidious onset , diffuse
  Dull , aching pain
- Exacerbated Forearm pronation

Anterior interosseous nerve
(Kiloh- Nevin syndrome)

- Clinical (o.k sign) + pain
- EDX

Iranian Congress OF Electrodiagnosis
IN
Peripheral Nerve Lesions
DEC.21.22/2006
Tehran-Iran

Anterior interosseus

- Neuralgic amyotrophy
- Forearm Fractures
- Injection
- Gun shot
- Elbow arthroscopy
- Laceration
- Pregnancy
- Gontzer ́s muscle

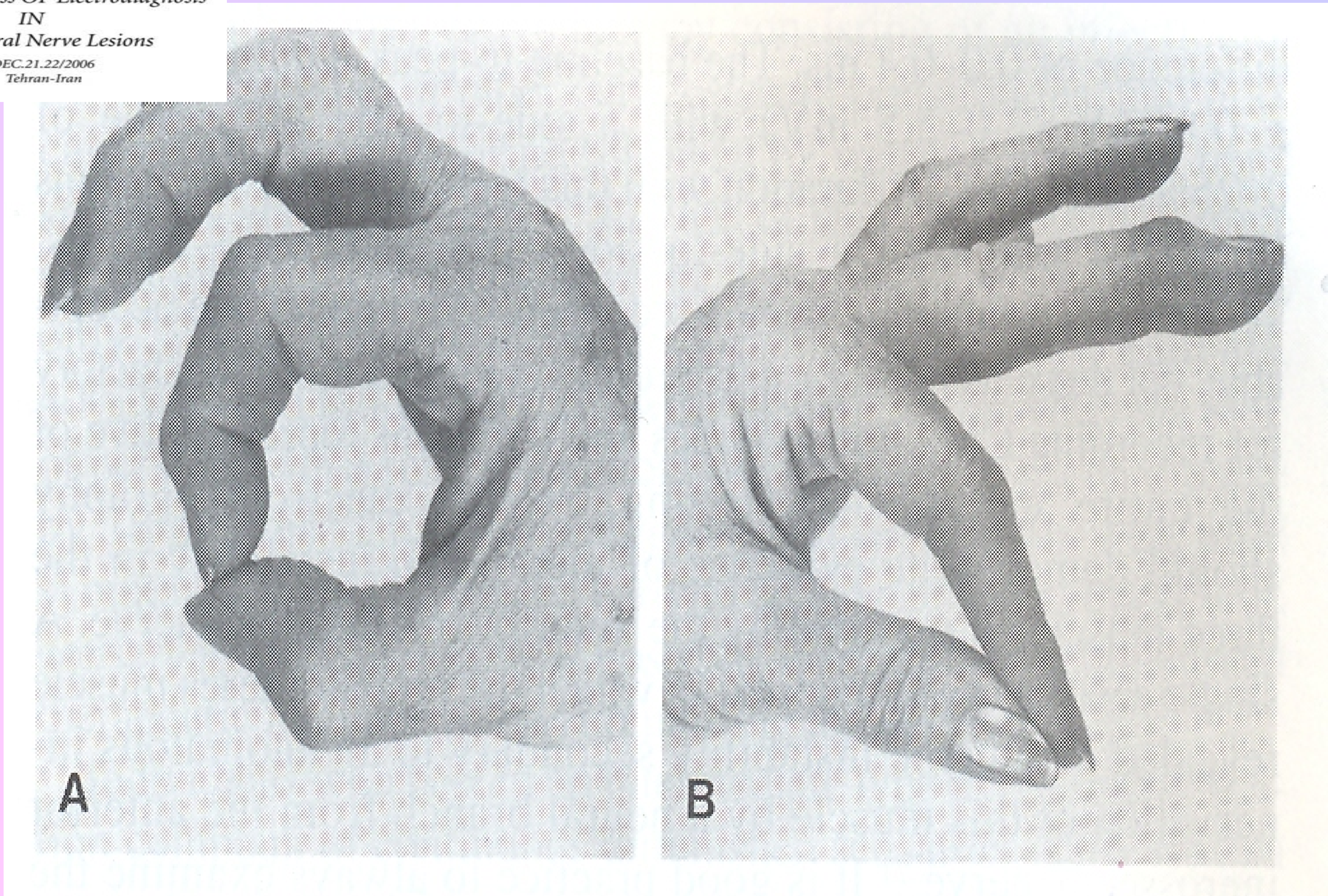
A
B

Iranian Congress OF Electrodiagnosis
IN
Peripheral Nerve Lesions
DEC.21.22/2006
Tehran-Iran

Anterior interosseus

- SNAP
- CMAP
- NEEDLE

Flexor pollisis longus
Pronator quadratus
Flexor digitorum profundus

Iranian Congress OF Electrodiagnosis
IN
Peripheral Nerve Lesions
DEC.21.22/2006
Tehran-Iran

Anterior interosseus?
Martin-Gruber?
