# Supplementary material for: Meeting report on the first Iranian congress of electrodiagnosis in peripheral nerve lesions
Source: J Brachial Plex Peripher Nerve Inj. 2007 Apr 14;2:10. doi: 10.1186/1749-7221-2-10 (PMC1865540; doi:10.1186/1749-7221-2-10)
Supplement: Additional file 1 — Slides from the invited lectures and panel discussions. Compressed PDFs of 15 presentations and 2 panel discussions during the conference. [file 1749-7221-2-10-S1.zip › radial nerve lesion.pdf]

Iranian Congress OF Electrodiagnosis
IN
Peripheral Nerve Lesions
DEC.21.22/2006
Tehran-Iran

RADIAL NEUROPATHY

A review to localization

S.AKRAMI MD
EMG CLINIC
EMAM KHOMEINI HOSPITAL
TEHRAN UNIVERSITY

Iranian Congress OF Electrodiagnosis
IN
Peripheral Nerve Lesions
DEC.21.22/2006
Tehran-Iran

Anatomy of Radial Nerve at arm

Γ1

- First courses on medial side of the humerus
- Winds obliquely around the humerus in the spiral groove (**Musculospiral Nerve**)
- Passing between the *heads of triceps muscle* & *fibrous arch formed by attachments of lateral head*
- Pierces LIS, enters the anterior compartment

Anatomy of Radial Nerve at elbow

- Courses between distal part of the biceps and brachioradialis

- Divides into

  - a deep motor branch (PIN)

  - a superficial sensory branch (superficial radial nerve)

Radial nerve position in supination and pronation

In supination with a **bare area** of the proximal radius, the PIN comes to *lie against the periosteum* of the radius.

Iranian Congress OF Electrodiagnosis
IN
Peripheral Nerve Lesions
DEC.21.22/2006
Tehran-Iran

Muscular branches above the elbow

3-5 branches to triceps at variable sites from axilla to the spiral groove

- Brachioradialis

- ECRL & B

These muscles often receive *additional*, more *distal branches* from the
- radial nerve itself
- superficial radial nerve
- Posterior Interosseous Nerve

Sunderland S. Nerves and nerve injuries, 2nd ed. Edinburgh: Churchill Livingstone, 1978

Sensory branches

- Posterior cutaneous nerve of the arm
- Lower lateral cutaneous nerve of the arm (lateral brachial cutaneous nerve)
- Posterior cutaneous nerve of the forearm
- Superficial branch of radial nerve

Iranian Congress OF Electrodiagnosis
IN
Peripheral Nerve Lesions
DEC.21.22/2006
Tehran-Iran

Autonomous zone of the radial nerve

- There is no *autonomous zone* for the radial nerve

- A coin sized area at first web space between thumb and index

Iranian Congress OF Electrodiagnosis
IN
Peripheral Nerve Lesions
DEC.21.22/2006
Tehran-Iran

EVALUATION OF RADIAL NERVE MOTOR FUNCTION

- ELBOW EXTENSION
- WRIST EXTENSION
- FINGER EXTENSION

Iranian Congress OF Electrodiagnosis
IN
Peripheral Nerve Lesions
DEC.21.22/2006
Tehran-Iran

Localization of radial nerve injuries

	Elbow extension	Wrist extension	Finger extension
Axilla	–	–	–
Midarm	+	–	–
Elbow	+	± (radial deviation)	–

– :no motion, +:good motion, ±: present motion with some weakness

Iranian Congress OF Electrodiagnosis
IN
Peripheral Nerve Lesions
DEC.21.22/2006
Tehran-Iran

CLINICAL DIAGNOSIS IN PERIPHERAL NERVE INJURIES

- ***RECOGNITION of IMPAIRED FUNCTION***

- ***IDENTIFICATION OF THE INJURY SITE (LOCALIZATION)***

- ***DEFINITION of THE MOST LIKELY ETIOLOGY(DIFFERENTIAL DIAGNOSTIC LIST)***

- ***USE of HELPING PROCEDURE TO DETERMINE THE ETIOLOGY***

Iranian Congress OF Electrodiagnosis
IN
Peripheral Nerve Lesions
DEC.21.22/2006
Tehran-Iran

CLINICAL & EDX LOCALIZATION

- ***TRADITIONAL ANATOMY***
- ***BRANCHING PATTERN***
- ***ANATOMIC VARIATION***
- ***SELECTIVE FASCICULAR INVOLVEMENT***

CLASSIC CLINICAL LOCALIZATION OF PERIPHERAL NERVE LESION

- ***INVOVED MUSCLES***
- ***INVOLVED CUTANEOUS AREA***
- ***KNOWING THE ANATOMY***

Iranian Congress OF Electrodiagnosis
IN
Peripheral Nerve Lesions
DEC.21.22/2006
Tehran-Iran

FASCICULAR ORGANIZATION

- **CABLE STRUCTURE** (*STOFFEL* 1913)

  FIBERS REMAIN AS DISCRETE FASCICLES

- **PLEXIFORM or INTERMINGLING STRUCTURE** (*LANGLEY* et al,1917)

  FIBERS BRANCH,SPLIT & REJOIN

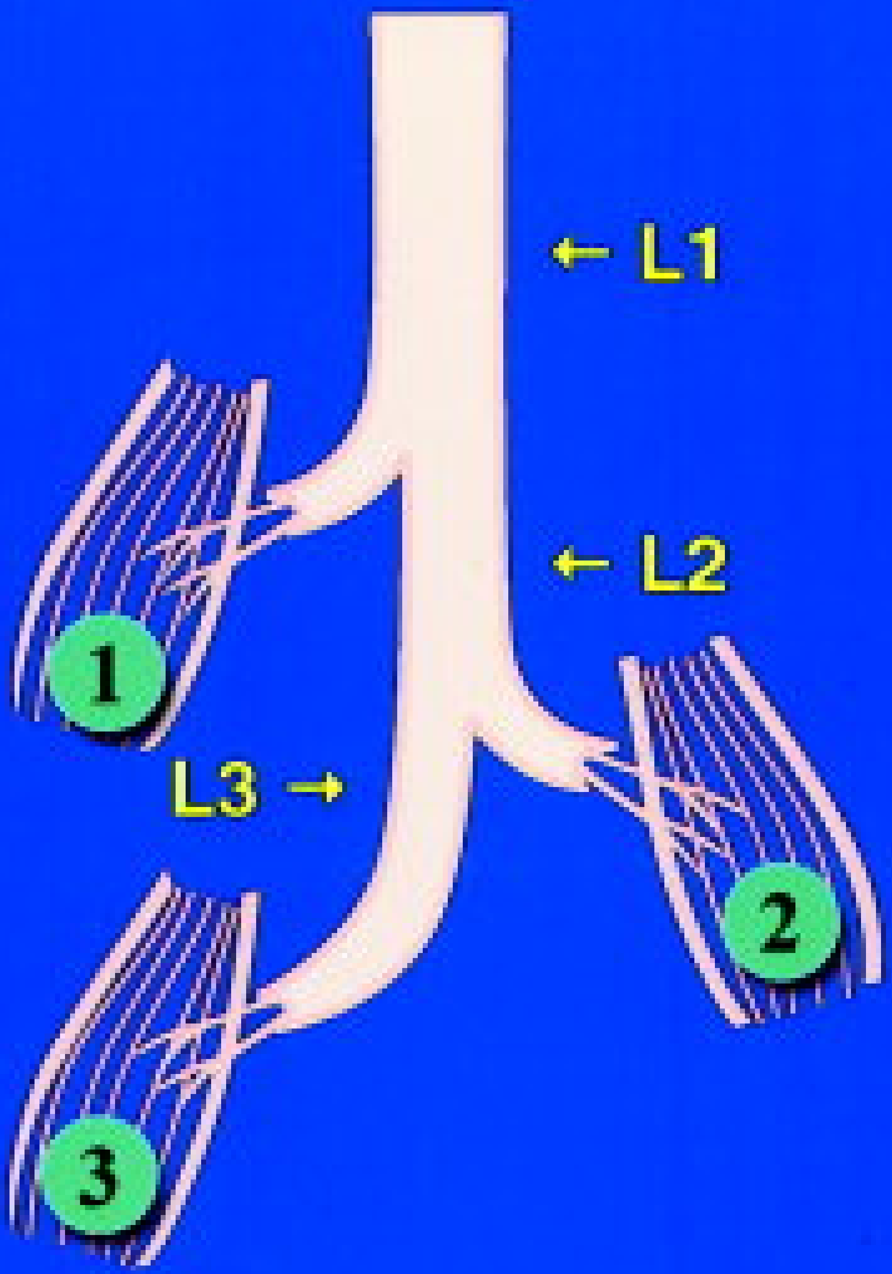
L1
L2
L3
1
2
3

THE INNERVATION PATTERN OF THE RADIAL NERVE AT THE ELBOW AND IN THE FOREARM

The most common branching pattern (from proximal to distal) was to

- brachioradialis
- extensor carpi radialis longus
- superficial sensory
- extensor carpi radialis brevis
- supinator
- extensor digitorum/extensor carpi ulnaris
- extensor digiti minimi
- abductor pollicis longus
- extensor pollicis brevis
- extensor pollicis longus
- extensor indicis

THE INNERVATION PATTERN OF THE RADIAL NERVE AT THE ELBOW AND IN THE FOREARM, G. BRANOVACKI, et al**,** *Journal of Hand Surgery (British and European Volume, 1998) 23B." 2:167-169*

Iranian Congress OF Electrodiagnosis
IN
Peripheral Nerve Lesions
DEC.21.22/2006
Tehran-Iran

THE INNERVATION PATTERN OF THE RADIAL NERVE AT THE ELBOW AND IN THE FOREARM

superficial radial

BB & ECRL

PIN

ECRB

Brachioradialis and **ECRL**

- The **brachioradialis** was the first forearm muscle to be innervated in 42 (70%) specimens.
- In 12 (20%) specimens the innervation of the brachioradialis and the ECRL came off at the same level .
- In six (10%) the ECRL came off proximal to the brachioradialis.

Innervation proximal to distal (right to left) of the brachioradialis, ECRL, and ECRB.

THE INNERVATION PATTERN OF THE RADIAL NERVE AT THE ELBOW AND IN THE FOREARM, G. BRANOVACKI, et al**,** *Journal of Hand Surgery (British and European Volume, 1998) 23B." 2:167-169*

Iranian Congress OF Electrodiagnosis
IN
Peripheral Nerve Lesions
DEC.21.22/2006
Tehran-Iran

Superficial sensory nerve

- The **superficial sensory branch** of the radial nerve originated

  - at the *same level* or
  - *distal to the ECRL*
  - but *proximal to the ECRB*

  in all specimens.

- One specimen showed an unusual variant of the superficial sensory nerve. The specimen had two branches of the superficial radial nerve originating off the radial nerve and running to the dorsum of the hand

This specimen shows two superficial sensory nerves.
Single arrow: origin.
Double arrow: distal continuation of two nerves.

THE INNERVATION PATTERN OF THE RADIAL NERVE AT THE ELBOW AND IN THE FOREARM, G. BRANOVACKI, et al, *Journal of Hand Surgery (British and European Volume, 1998) 23B." 2:167-169*

Extensor carpi radialis brevis

- There was a single branch to the **ECRB** in all specimens, but the point of origin of this branch varied considerably.

- The **nerve branch to the ECRB** muscle arose from:

- the *posterior interosseous nerve* in 27 (45%)

- at the *bifurcation* of posterior interosseous and superficial sensory nerves in 18 (30%) of the specimens and

- from the *superficial sensory* nerve in 15 (25%)

Supinator

- The **innervation** of the supinator was also found to be highly variable.
- The supinator frequently had *multiple innervating branches*.
- All of the specimens had **innervating branches** to the supinator originating proximal to the muscle.
- Twelve specimens (20%) had a branch that arose proximal to the *joint*.
- The **PIN** gave off a branch to the supinator while passing through the muscle in seven specimens (12%).

Iranian Congress OF Electrodiagnosis
IN
Peripheral Nerve Lesions
DEC.21.22/2006
Tehran-Iran

APL, EPB, EPL, EI

- The **APL branch** came off
  - *proximal to the EPB* branch in 21 (35%) specimens and
  - the **APL** and **EPB** came off as a common stem in 39 (65%).

- the **EPL branch** arose
  - *before the EI branch*, In 37 (62%) specimens
  - from the *same branch*, in 20 (33%)
  - and in three (5%) the branch to the *EI arose proximal to the branch to the EPL*

- Either the **EPL** or the **EI** had a very distal nerve branch 6 to 7 cm distal to the first innervating branch in 28 (47%) specimens.

Iranian Congress OF Electrodiagnosis
IN
Peripheral Nerve Lesions
DEC.21.22/2006
Tehran-Iran

Five potential compression sites of the PIN from proximal to distal

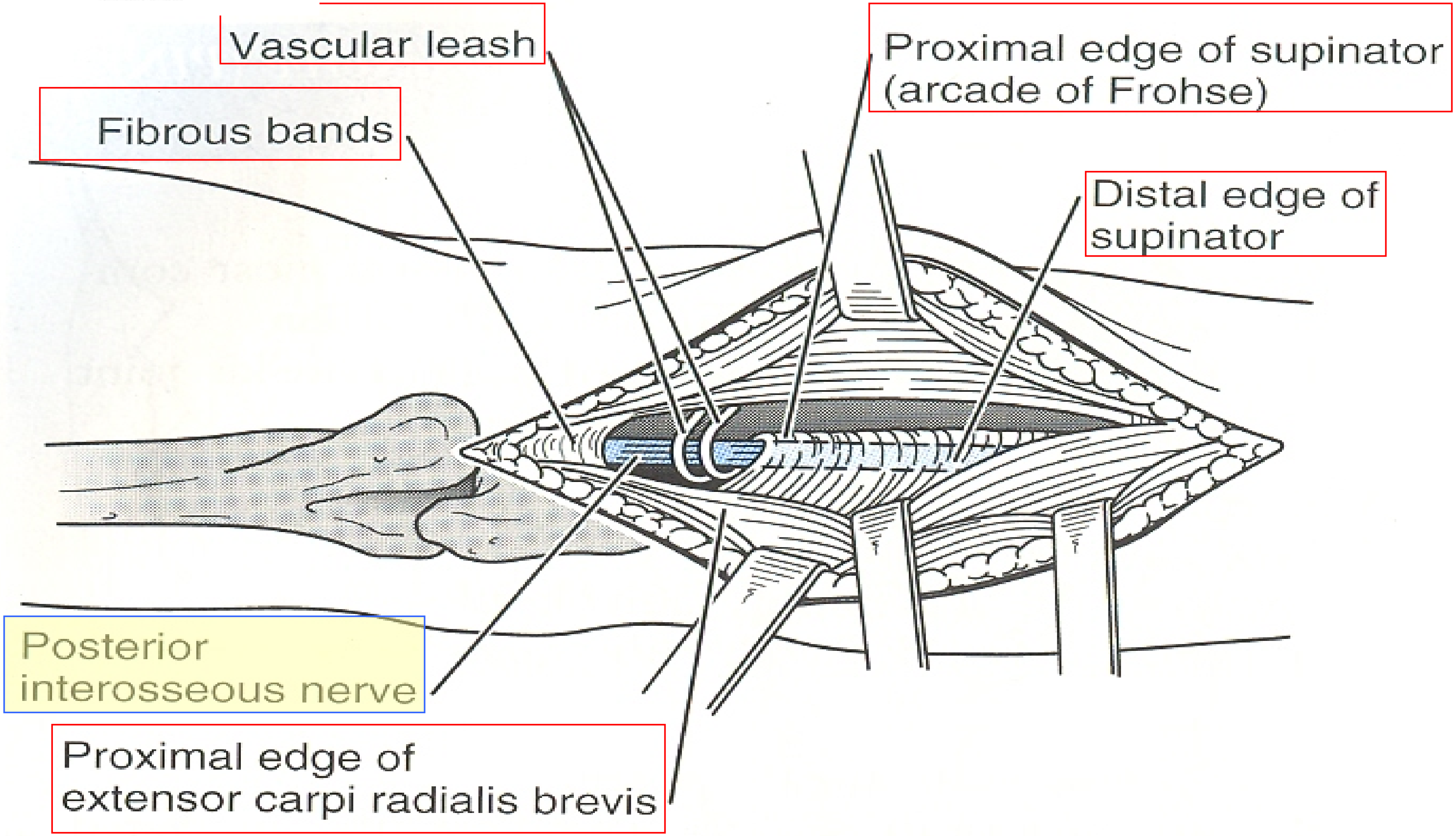

Iranian Congress OF Electrodiagnosis
IN
Peripheral Nerve Lesions
DEC.21.22/2006
Tehran-Iran

Conclusions

- Radial neuropathy may occur at different points (axilla, arm, elbow, forearm).
- Variable clinical motor & sensory signs may be observed.
- Variable innervation branching can cause different clinical and Edx patterns.
- Internal topography (**fascicular anatomy**) and **innervation patterns** are important for localization.
