# Supplementary material for: Meeting report on the first Iranian congress of electrodiagnosis in peripheral nerve lesions
Source: J Brachial Plex Peripher Nerve Inj. 2007 Apr 14;2:10. doi: 10.1186/1749-7221-2-10 (PMC1865540; doi:10.1186/1749-7221-2-10)
Supplement: Additional file 1 — Slides from the invited lectures and panel discussions. Compressed PDFs of 15 presentations and 2 panel discussions during the conference. [file 1749-7221-2-10-S1.zip › REPORT GENERATION IN EDX.pdf]

Iranian Congress OF Electrodiagnosis
IN
Peripheral Nerve Lesions
DEC.21.22/2006
Tehran-Iran

بنام خدا

Iranian Congress OF Electrodiagnosis
IN
Peripheral Nerve Lesions
DEC.21.22/2006
Tehran-Iran

Report Generation
in

The Electrodiagnostic Medicine Consultation

Dr. M. Baghbani
Physiatrist

Iranian Congress OF Electrodiagnosis
IN
Peripheral Nerve Lesions
DEC.21.22/2006
Tehran-Iran

Contents

Consultation Form

Report Form

- History
- Physical Examination
- Nerve Conduction Studies Table
- Needle EMG Table
- Summary of Findings
- Impression
- Recommendation
- Copies of Waveforms

Iranian Congress OF Electrodiagnosis
IN
Peripheral Nerve Lesions
DEC.21.22/2006
Tehran-Iran

Consultation Form

* Local Problem (sharp injury)
* Explicitly Problem (Rt hand numbness)
* Limb Problem (Radicular Pain)
* Generalized Problem (gait, weakness , ...)

The Referring physicians request is important , this is *guide for EDX evaluation*

Iranian Congress OF Electrodiagnosis
IN
Peripheral Nerve Lesions
DEC.21.22/2006
Tehran-Iran

Report Form

Patients chief complaint

↓

Directed Hx & physical Exam

↓

Comperhensive Focused EDX evaluation

↓

Data Analyzed

↓

Findings Communication

↙ ↘

Impression | **Recommendation**

Iranian Congress OF Electrodiagnosis
IN
Peripheral Nerve Lesions
DEC.21.22/2006
Tehran-Iran

Physical medicine
&
Rehabilitation center

Electrodiagnostic studies report

Iranian Congress OF Electrodiagnosis
IN
Peripheral Nerve Lesions
DEC.21.22/2006
Tehran-Iran

Electrodiagnostic Examination Report continue

Name : Age: Date: Referring Physician :

History & Physical Exam :

Referral diagnosis :

Iranian Congress OF Electrodiagnosis
IN
Peripheral Nerve Lesions
DEC.21.22/2006
Tehran-Iran

Sensory nerve conduction studies

Side	Nerve	DL (m.sec)	Amp	NCV	Explanation
Rt	Median	6.5	10	28	Latente, low Amp

Motor nerve conduction studies & H,F wave

side	nerve	DL	Amp	PL	Amp	NCV	F.wave	H.reflex
Rt	ulnar	2.9	6	5	5.8	54	21	-

Iranian Congress OF Electrodiagnosis
IN
Peripheral Nerve Lesions
DEC.21.22/2006
Tehran-Iran

Repetitive Stimulation Test

Side	nerve	Muscle	Pre. Exe Dec	Imm. post Exe Dec	1.5 min post Exe Dec	Jitter	Fiber density

Iranian Congress OF Electrodiagnosis
IN
Peripheral Nerve Lesions
DEC.21.22/2006
Tehran-Iran

Electromyography

muscle	Nerve	Root	Rest Activity			Volition		Atypic potential
			IA	P.S.W	Fib	Rec	IF	

Abrevation : IA: Insertional Activity P.S.W: Positive Sharp wave FIB: Fibrilation
DE: Decreased NL: normal IN: Increased MUAP: Motor Unit Action potential
IF : interfrence Rec : Recruitment

Iranian Congress OF Electrodiagnosis
IN
Peripheral Nerve Lesions
DEC.21.22/2006
Tehran-Iran

Summary of findings :

EDX Impression :

Clinical Impression :

Recommendation :

Physical medicine & Rehabilitation center

Electrodiagnostic studies report

Name : Age: Date: Referring Physician :
History & Physical Exam :
Referral diagnosis :

Sensory nerve conduction studies

Side	Nerve	DL (m.sec)	Amp	NCV	Explanation
Rt	Median	6.5	10	28	

Motor nerve conduction studies & H,F wave

side	nerve	DL	Am	PL	Amp	NCV	F.wave	H.refle
Rt	ulnar	2.9	P 6	5	5.8	54	21	x -

Iranian Congress OF Electrodiagnosis
IN
Peripheral Nerve Lesions
DEC.21.22/2006
Tehran-Iran

Electromyography

muscle	Nerve	Root	Rest Activity			Volition		Atypic.p
			IA	P.S.W	Fib	Rec	IF	

Abrevation : IA: Insertional Activity P.S.W: Positive Sharp wave FIB: Fibrilation
DE: Decreased NL: normal IN: Increased MUAP: Motor Unit Action potential

Summary of findings :

EDX impression :

Clinical impression :

Recommendation :

Iranian Congress OF Electrodiagnosis
IN
Peripheral Nerve Lesions
DEC.21.22/2006
Tehran-Iran

Example : I

Referring diagnosis: Brachial Plexus injury

EDX impression : C8-T1 Roots avulsion

Clinical impression : myofascial pain syndrome

Recommendation :

* OT
* pain clinic

Iranian Congress OF Electrodiagnosis
IN
Peripheral Nerve Lesions
DEC.21.22/2006
Tehran-Iran

Example : 2

Referring Diagnosis :

ulnar neuropathy

EDX impression :

complete wallerian degeneration

Clinical impression :

____

Recommendation :

follow-up on 6 month later for ...

Iranian Congress OF Electrodiagnosis
IN
Peripheral Nerve Lesions
DEC.21.22/2006
Tehran-Iran

thanks for your attention
