# Supplementary material for: Meeting report on the first Iranian congress of electrodiagnosis in peripheral nerve lesions
Source: J Brachial Plex Peripher Nerve Inj. 2007 Apr 14;2:10. doi: 10.1186/1749-7221-2-10 (PMC1865540; doi:10.1186/1749-7221-2-10)
Supplement: Additional file 1 — Slides from the invited lectures and panel discussions. Compressed PDFs of 15 presentations and 2 panel discussions during the conference. [file 1749-7221-2-10-S1.zip › ROOTS LESION.pdf]

Iranian Congress OF Electrodiagnosis
IN
Peripheral Nerve Lesions
DEC.21.22/2006
Tehran-Iran

Radiculopathy

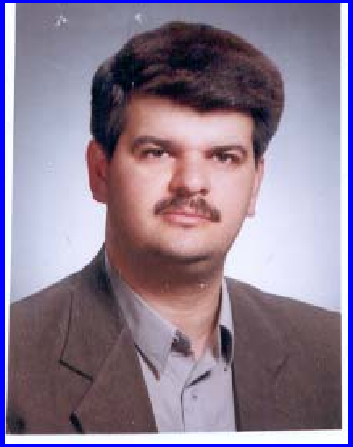

BIJAN FOROGH , MD

Dept of PM&R
Iran University of Medical Science

bijhanfr@yahoo.com
Tel: 22221577

Objectives

- Anatomy
- Pathophysiology
- Clinical presentation and assessment
- Differential diagnosis
- Investigations
- Radiculopathies by spinal region
- Special situations

Spinal Neural Anatomy

- Spinal cord
- Nerve roots
- Dorsal root ganglion
- Spinal nerve
- Dorsal & ventral rami

Spinal Neural Anatomy

- Spinal nerve & DRG located in intervertebral foramen at most levels
- Cervical levels: nerve & DRG extend to sulcus of transverse process
- Sacral levels: nerve & DRG remain in sacral canal
- Dorsal ramus: innervate paraspinal structures
- Ventral ramus: innervate limb and anterolateral trunk muscles and skin

Iranian Congress OF Electrodiagnosis
IN
Peripheral Nerve Lesions
DEC.21.22/2006
Tehran-Iran

Spinal Neural Anatomy

- Length discrepancy between vertebral column and spinal cord
- Spinal cord ends at L2-3 level from 5 years of age
- Cervical roots travel horizontally to their foramen
- Lumbosacral roots descend vertically to their foramen (cauda equina)

Cervical Neural Anatomy

- 7 cervical vertebrae, 8 spinal nerves
- C1- C7 roots exit spinal canal above their corresponding vertebrae
- C8 root exits between C7 and T1 vertebrae

Iranian Congress OF Electrodiagnosis
IN
Peripheral Nerve Lesions
DEC.21.22/2006
Tehran-Iran

Thoracic-lumbosacral Neural Anatomy

- All thoracic and lumbosacral roots exit below their corresponding vertebrae
- Roots exit in superior portion of foramen, superior to disc

Paraspinal Muscles

- Superfical and deep muscle layers
- Deep paraspinal muscles: erector spinae, semispinalis, rotators and multifidi
- Multifidi
  - Most medial of deep back muscles
  - Unisegmental innervation by posterior rami
  - Ideal muscle for needle placement

Iranian Congress OF Electrodiagnosis IN Peripheral Nerve Lesions
DEC.21.22/2006
Tehran-Iran

Myotomes & Dermatomes

- Myotome
  - Muscles innervated by the same spinal cord segment and root
  - Most muscles are innervated by 2-3 segments or roots
- Dermatome
  - Skin region innervated by a spinal cord segment or root
  - Significant overlap between dermatomes

Pathophysiology of radiculopathy

- Neural compression
  - Disc protrusion/rupture
  - Degenerative changes
  - Others
- Inflammation
- Vascular compromise

Pathophysiology

- Generally extrapolated from peripheral nerve injury knowledge
- Root anatomy different from peripheral nerve
- Focal demyelination
  - Conduction block
  - Slowing of conduction velocity
- Axonal injury
  - Denervation of muscle fibers
  - Sensory pathway degeneration

Clinical Presentation

- Sensory symptoms most common
- Pain
- Numbness
- Paresthesias
- Weakness

Clinical Assessment

- Spine & neurological exam
  - Sensory testing
  - Muscle strength
  - Reflex changes & symmetry
  - Decreased spinal ROM
  - Antalgic movement patterns

Iranian Congress OF Electrodiagnosis IN Peripheral Nerve Lesions
DEC.21.22/2006
Tehran-Iran

Root tension tests

- Straight leg raise test & modifications
- Cross SLR test
- Reverse SLR test
- Spurlings test

Clinical assessment of suspected radiculopathy

- An abnormal electrodiagnostic study is 2-4X more likely when at least one abnormal examination finding is present
- Abnormal electrodiagnostic studies can be present with normal physical examinations

Lauder TD. Physical examination signs, clinical symptoms, and their relationship to electrodiagnostic findings and the presence of radiculopathy. PM&R Clinics Aug 2002.

Differential Diagnosis

- Focal/generalized neuropathies
- Plexopathies
- Anterior horn cell disorders
- Musculoskeletal disorders
- Myofascial pain syndrome
- Referred pain

Electrodiagnostic Studies

- Nerve conduction studies
- Needle electromyography
- Late responses
  - H-reflex
  - F wave
- Somatosensory evoked potentials
- Nerve root stimulation

Needle EMG

- Most established electrodiagnostic method
- Highest diagnostic yield
- Assesses motor component
- EMG abnormalities
  - Abnormal spontaneous activity
  - Increased insertional actiivity
  - Recruitment abnormalities
  - Motor unit changes

Needle EMG

- Diagnosis by identification of EMG abnormalities in a myotomal distribution
- 2 or more muscles innervated by a common root and separate peripheral nerves
- Lack of abnormalities in adjacent myotomes

Needle EMG abnormalities

- Axonal injury to motor fibers
  - Abnormal spontaneous activity
  - Increased insertional activity
  - Reduced motor unit recruitment
  - Motor unit morphological changes from reinervation
- Focal demyelination
  - Reduced recruitment

Time course of EMG abnormalities

- Sequence of appearance of needle EMG abnormalities time dependent
- Guide to chronicity of lesion
- Explains presence/absence of electrodiagnostic abnormalities

Time course of EMG abnormalities

- PSWs/fibrillation potentials
  - First present in proximal muscles-paraspinals (7 days)
  - Later appearance in distal muscles (3-5 weeks)
  - Decreased numbers and amplitudes with time (<100 microV)
  - Can also disappear after reinnervation

Time course of EMG abnormalities

- Fasiculations
  - Uncommon
  - Appear after 1- 6 weeks
- Complex Repetitive Discharges
  - Uncommon
  - Late appearance (> 3 mths)

Time course of EMG abnormalities

- 1st week : Reduced MUP recruitment
- Weeks – months: MUP waveform changes from renervation
- Quantitative EMG analysis
- EMG findings are dependent on the balance between muscle fiber denervation and reinnervation

EMG screen for cervical radiculopathy

- Assuming a root lesion is diagnosable by EMG, how many muscles need to be examined to obtain high diagnostic yields?
- 6 muscle screens (including paraspinals) detected 94%-99% of cervical radiculopathies
- 8 limb muscles (without parapspinals) detected 92%-95% cervical radiculopathies

Dillingham T et al. Identification of Cervical Radiculopathies: Optimizing the Electromyographic Screen. Am J Phys Med Rehabil 2001; 80(2) 84-91.

EMG screen for L-S radiculopathy

- 6 muscle screens (including paraspinals) identified 98%-100% of L-S radiculopathies
- 8 limb muscles (without paraspinals) required to detect 87%-90% of L-S radiculopathies

Dillingham T et al. Identifying Lumbosacral Radiculopathies: An Optimal Electromyographic Screen. Am J Phys Med Rehabil 2000; 79(6) 496-503.

Paraspinal muscle examination

- Paraspinal muscle assessment increases diagnostic yield
- Localizes lesion to root level or proximal to plexus
- Some (20%-40%) patients only have abnormalities in paraspinal muscles

Paraspinal muscle examination

- Paraspinal EMG can be abnormal in pathology of anterior horn cells, posterior rami or muscle
- Paraspinal muscle EMG can be normal in radiculopathy

Nerve Conduction Studies

- Should do at least 1 motor and sensory nerve study in symptomatic limb
- Helps rule out other pathology

Iranian Congress OF Electrodiagnosis
IN
Peripheral Nerve Lesions
DEC.21.22/2006
Tehran-Iran

Nerve Conduction Studies

- Sensory nerve studies
  - C6-thumb, index finger, C7-3rd finger, C8-5th finger
  - L4-saphenous, L5 sup. peroneal, S1-sural
- Generally normal in radiculopathies
- Site of root lesion usually proximal to DRG
- Decreased SNAP amplitudes occasionally

Nerve Conduction Studies

- Motor nerve studies
- Usually normal in single radiculopathies
  - Muscles innervated by more than one root
  - Incomplete axonal lesions or conduction block
- CMAP amplitudes can be reduced in severe axonal loss or in multi-root involvement

H-Reflex

- May be helpful in S1 and C6/7 radiculopathies
- Record over soleus and FCR
- Assesses proximal sensory fibers and motor fibers
- Immediate onset of abnormality

H-Reflex

- Can be normal in radiculopathy
- When abnormal, test does not localize site of lesion
- Abnormal reflex can persist
- Wide range of normal values
- CNS modulation

F-Wave

- Can assess proximal portion of PNS
- Examines motor fibers only
- Poor localization of abnormality
- Diagnostic criteria often non-standardized
- Often normal in single root or partial root involvement

Somatosensory evoked potentials

- Assesses sensory fibers from site of stimulation to sensory cortex
- Site of stimulation
  - Mixed nerve
  - Sensory nerve
  - Dermatome
- Diagnostic criteria varies

Iranian Congress OF Electrodiagnosis
IN
Peripheral Nerve Lesions
DEC.21.22/2006
Tehran-Iran

Somatosensory evoked potentials

- Caveats
  - Wide range of normal values for amplitude & latency and for side to side differences
  - Meticulous technique and time required
  - Poor localization of root lesion
  - Diagnostic yields have varied from study to study
  - Does not improve on routine NCVs/EMG

Nerve Root Stimulation

- Stimulate nerve root and record evoked motor potential from innervated muscle
- Root stimulation with needles or magnetic coils
- Side to side comparisons of CMAP latency, amp, area

Limitations

- Assesses only motor fibers
- Difficult to stimulate only single roots
- Exact stimulation site unclear i.e. proximal or distal to neural foramen

Iranian Congress OF Electrodiagnosis
IN
Peripheral Nerve Lesions
DEC.21.22/2006
Tehran-Iran

Advantages of EMG/NCVs

- Assesses physiological status of roots
- Low rate of false positives in good hands
- Can identify other peripheral nerve disorders
- Low morbidity

Limitations

- Needle EMG cannot detect:
  - sensory fiber involvement
  - limited motor fiber involvement
  - Slow progressive lesions
- Misinterpretation of EMG findings
- Technical difficulties i.e. thoracic paraspinals
- Not assessing enough muscles or nerve

Limitations

- Overinterpretation of findings
  - Overcalling MUAP changes without quantitative analysis i.e. polyphasics
  - Diagnosing solely on recruitment and MUAP changes

Limitations

- Confounding co-existing conditions i.e. neuropathies
- Inability of routine NCS to assess small diameter fibers

Imaging Studies

- Plain radiographs
- CT imaging
- Myelogram
- MRI

Imaging Studies

- All imaging studies i.e. MRI, CT, myelography assess structural anatomy
- Problem: often show “abnormalities” in asymptomatic normal subjects or spinal regions

EMGs vs Imaging studies

- EMG/NCVs
  - Evaluates nerve function & detects pathophysiology
- Imaging studies – MRI, CT
  - Evaluates structure and detects “structural abnormalities”

EMG vs Imaging

- Sensitivity of EMG relative to clinical, imaging or surgically diagnosed radiculopathy varies from 49% to 92%
- EMG likely to have higher specificity
- Can help determine clinical relevance of ‘abnormalities” discovered on imaging and diagnose other disorders

EMG vs Imaging

- Comparison of EMG and MRI
- Agreement between EMG & MRI in 60% subjects
- 40% of subjects had one abnormal test
- Suggests studies are complementary

Nardin RA. Electromyography and magnetic resonance imaging in the evaluation of radiculopathy. Muscle Nerve 1999;22(2):151-155.

Diagnostic selective nerve root blocks

- May be helpful when
  - imaging and EMG are non-diagnostic, or in patients with suspected radiculitis, failed back surgery or anomalous anatomy
  - Failure to improve with conservative care
- Identification of specific pain generator (nerve root)
- Demonstration of significant pain relief with deposition of local anesthetic around the spinal nerve and DRG under fluoroscopic guidance

Cervical Radiculopathies

- Second most common spinal region for radiculopathies
- Frequency of affected roots
  - C7>C6>C8>C5
- Majority (70%-90%) are single root lesions

Cervical Radiculopathies

- C 3/4 roots
  - Pain and paresthesia of posterolateral scalp
  - Greater and lesser occipital nerves
  - Great auricular nerve
  - EDx not used

Cervical Radiculopathies

- C5 root
  - Often from C4-C5 disc
  - Abnormalities in rhomboid, supra/infraspinatus, deltoid, biceps, brachialis, brachioradialis
  - Can be difficult to differentiate from C6 radiculopathy due to common muscle innervations

Cervical Radiculopathies

- C6 root
  - Many common muscles with C5 root
  - Pronator teres, extensor/ flexor carpi radialis abnormalities point to C6 lesion
  - Abnormal FCR H-reflex

Cervical Radiculopathies

- C7 root
  - Abnormalities in triceps, anconeus, flexor carpi radialis, pronator teres
  - Assess both radial & median innervated muscles
  - Abnormal H-reflex

Cervical Radiculopathies

- C8 root
  - Shares innervation of many muscles with T1 root
  - Abnormalities in all ulnar nerve innervated muscles, EIP, EPB (radial), FPL (median)

Multiple Radiculopathies

- Most cervical radiculopathies from disc lesions involve single roots
- When multiple radiculopathies detected, should assess contralateral limb
- When both upper limbs are abnormal, assess lower limbs

Lumbosacral Radiculopathies

- Most frequent radiculopathies
- L5 & S1 roots most commonly affected
- Most disc herniations are posterolateral
- Root exiting at next caudal level affected i.e L4-5 disc and L5 root
- Single lumbar disc can compress multiple roots depending on direction and size of herniation

Lumbosacral Radiculopathies

- L 2,3,4 roots
- Abnormalities in quadriceps, thigh adductors, iliopsoas
- Differentiation from peripheral nerve and plexus lesions can be difficult i.e diabetic amyotrophy

Iranian Congress OF Electrodiagnosis
IN
Peripheral Nerve Lesions
DEC.21.22/2006
Tehran-Iran

Lumbosacral Radiculopathies

- L5 root
- Most common single radiculopathy
- Abnormalities in peroneal nerve muscle
  - tibialis ant. extensor hallucis, peroneus longus , EDB
- Abnormalities in tibial nerve muscles
  - flex dig longus, tibialis post., hamstrings
- Abnormalities in non-sciatic nerve muscles
  - TFL and glutei

Lumbosacral Radiculopathies

- Foot drop
- DDx: peroneal nerve lesion, disorders of motor neuron
- Test inversion
- Post. tibial muscle spared in peroneal lesions

Iranian Congress OF Electrodiagnosis
IN
Peripheral Nerve Lesions
DEC.21.22/2006
Tehran-Iran

Lumbosacral Radiculopathies

- S1 root
- 2nd most common single radiculopathy
- Often bilateral
- Abnormalities in foot muscles, gastrosoleus, glutei, hamstrings
- H reflex can be abnormal

Iranian Congress OF Electrodiagnosis IN Peripheral Nerve Lesions
DEC.21.22/2006
Tehran-Iran

Lumbosacral Radiculopathies

- S 2,3,4 roots
- Multiple & bilaterally involvement in cauda equina lesions
- Bowel, bladder and sexual disturbance
- Cauda equina syndrome: neurosurgical emergency
- Abnormalities in abductor hallucis and abductor of 5th toe, external anal sphincter

Iranian Congress OF Electrodiagnosis IN Peripheral Nerve Lesions
DEC.21.22/2006
Tehran-Iran

Lumbar Spinal Stenosis

- 92% of patients with spinal stenosis confirmed by imaging had a positive EMG
- 46% of patients with positive EMG did not have paraspinal muscle abnormalities
- 76% of patients had bilateral myotomal findings

Hall et al. Lumbar spinal stenosis. Ann Intern Med 1985;103:271-275.

Thoracic Radiculopathies

- Uncommon occurrence
- Disc pathology less common
- Diabetes and herpes zoster
- Electrodiagnosis technically challenging

Special Situations

- Post spinal surgery & procedures
- Medical causes of radiculopathy
  - Diabetes
  - Infection
  - Cancer

Summary

- Spinal anatomy
- Etiology & pathophysiology
- Clinical presentation
- Physical examination
- Investigations
- Electrodiagnostic testing
- Distribution of radiculopathies

Iranian Congress OF Electrodiagnosis
IN
Peripheral Nerve Lesions
DEC.21.22/2006
Tehran-Iran

SELECTED BIBLIOGRAPHY

- American Association of Electrodiagnostic Medicine. Guidelines in electrodiagnostic medicine. Muscle Nerve 1999; Suppl 8:S5-300.
- Dillingham TR. Electrodiagnostic approach to patients with suspected radiculopathy. In Kraft GH, Dillingham TR (eds) Update in Diagnosis and Management of Radiculopathies. Physical Medicine and Rehabilitation Clinics of North America. Philadelphia, WB Saunders Company, August 2002, 567-588.
- Dillingham TR, Lauder TD, Andary M, Kumar S, Pezzin LE, Stephens RT, et al. Identification of cervical radiculopathies: optimizing the electromyographic screen. Am J Phys Med Rehabil 2001;80: 84-91.

Iranian Congress OF Electrodiagnosis
IN
Peripheral Nerve Lesions
DEC.21.22/2006
Tehran-Iran

SELECTED BIBLIOGRAPHY

- Dillingham TR, Lauder TD, Huston CW, Chang AS, Belandes PV. Lumbosacral root screen:optimizing the number of muscles studied. Am J Phys Med Rehabil 1994;73:394-402.
- Dumitru D, Zwarts MJ. Radiculopathies. In Dumitru D, Amato AA, Zwarts MJ (eds): Electrodiagnostic Medicine, 2nd ed. Philadelphia, Hanley & Belfus, Inc, 2002, 713-776.
- Hall S, Bartleson JD, Onofrio BM, Baker HL Jr, Okazaki H, O’ Duffy JD. Lumbar spinal stenosis: clinical features, diagnostic procedures, and results of surgical treatment in 68 patients. Ann Intern Med 1985;103;271-275.
- Johnson EW, Burkhart JA, Earl WC. Electromyography in post laminectomy patients. Arch Phys Med Rehabil 1972; 53:407-409.

SELECTED BIBLIOGRAPHY

- Kimura J. Radiculopathies and plexopathies. In Kimura J: Electrodiagnosis in diseases of nerve and muscle: principles and practice, 3rd ed. New York, Oxford University Press, Inc, 2001, 628-649.
- Lauder TD. Physical examination signs. Clinical symptoms, and their relationship to electrodiagnostic findings and the presence of radiculopathy. In Kraft GH, Dillingham TR (eds) Update in Diagnosis and Management of Radiculopathies. Physical Medicine and Rehabilitation Clinics of North America. Philadelphia, WB Saunders Company, August 2002, 451-485.

Iranian Congress OF Electrodiagnosis
IN
Peripheral Nerve Lesions
DEC.21.22/2006
Tehran-Iran

SELECTED BIBLIOGRAPHY

- Nardin RA, Patel MR, Gudas TF, Rutkove SB, Raynor EM. Electromyography and magnetic resonance imaging in the evaluation of radiculopathy. Muscle Nerve 1999;22:151-155
- Wilbourn AJ, Aminoff MJ. AAEM Minomonograph #32. The electrophysiologic examination in patients with radiculopathies. Muscle Nerve 1998; 21: 1612-1631.
