# Supplementary material for: Meeting report on the first Iranian congress of electrodiagnosis in peripheral nerve lesions
Source: J Brachial Plex Peripher Nerve Inj. 2007 Apr 14;2:10. doi: 10.1186/1749-7221-2-10 (PMC1865540; doi:10.1186/1749-7221-2-10)
Supplement: Additional file 1 — Slides from the invited lectures and panel discussions. Compressed PDFs of 15 presentations and 2 panel discussions during the conference. [file 1749-7221-2-10-S1.zip › SCIATIC NERVE LESIONS.pdf]

Iranian Congress OF Electrodiagnosis
IN
Peripheral Nerve Lesions
DEC.21.22/2006
Tehran-Iran

Sciatic Nerve Lesion

Dr Mansoori MD

Professor Associate Of Physical Medicine & Rehabilitation

iums

Iranian Congress OF Electrodiagnosis
IN
Peripheral Nerve Lesions
DEC.21.22/2006
Tehran-Iran

Type Of Injuries

- Proximal Lesions
- Open

  Gunshot Wounds

  Knife Wounds

  Laceration

  Injection

  Surgery Related (Hip Replacement)

- Closed

pelvic Fractures

Acetabular Fracture/ Dislocation

Compression:

- External(Addiction-Drug-alcohol)

- Internal (tumor – hematoma)

- Distal Lesions
- Open

  Gunshot Wounds

  Knife Wounds

  Lacerations

  Femur Fractures

Iranian Congress OF Electrodiagnosis
IN
Peripheral Nerve Lesions
DEC.21.22/2006
Tehran-Iran

- Closed

Femural Fractures

Compression :

- External

- Internal (myositis ossificans-tumor)

Iranian Congress OF Electrodiagnosis
IN
Peripheral Nerve Lesions
DEC.21.22/2006
Tehran-Iran

Injection Injury

- Direct Puncture Of Nerve
- Placement Of Neurotoxic Agent
- Delayed Reaction & Scar
- Delayed Symptoms if A hematoma Formed
- Reflex Sympathetic Dystrophy

Iranian Congress OF Electrodiagnosis
IN
Peripheral Nerve Lesions
DEC.21.22/2006
Tehran-Iran

More Peroneal Injury

- Lateral Position
- Large Fascicles
- Less Interestitial Tissue
- Fixation To The Notch

Iranian Congress OF Electrodiagnosis
IN
Peripheral Nerve Lesions
DEC.21.22/2006
Tehran-Iran

Piriformis syndrom

- H Findings
- Involvment Below Gluteal Area
- No Gluteal & Paravertebral Involvment
- Clinical Findings

Iranian Congress OF Electrodiagnosis
IN
Peripheral Nerve Lesions
DEC.21.22/2006
Tehran-Iran

Sciatic Injury At Thigh

- Less Frequent
- Missile Fragments
- Fractures
- Lacerations
- Compression
- Tumors
- Myositis Ossificans

Iranian Congress OF Electrodiagnosis
IN
Peripheral Nerve Lesions
DEC.21.22/2006
Tehran-Iran

Clinical Examination

- Gluteal Muscle Examination
- Full Sensory Examination
- Reflexes
- Foot plantar & dorsiflexors
- Ankle invertor & evertors
- Hamstring examination(Large Muscle Miss Weakness)
- Quadriceps examination
- Anal Sphincter

Iranian Congress OF Electrodiagnosis
IN
Peripheral Nerve Lesions
DEC.21.22/2006
Tehran-Iran

Electrodiagnostic Evaluations

- CMAP From EDB & AHB Or Proximals
- Above & Below Fibular Head
- H & F Waves Are Necessory But Not Specific
- SNAP in separating root from other lesions & severity of axonal lesions
- Needle Electromyography Is Helpful In Level ,severity & Prognosis (Paravertebral-Glteal-Quadriceps-hamstrings-short head of biceps…)

  -Anal sphincter is also important

Iranian Congress OF Electrodiagnosis
IN
Peripheral Nerve Lesions
DEC.21.22/2006
Tehran-Iran

Never Forget

- Examination From Distal To Proximal
- Examine Under Splints & Orthoses
- Examine Above Castings
- Compare Two Sides Carefuly(Motor-Sensory-F & H Waves) Present Waves Are Not Indicative Of Normality
- Examine Every Thing Come To Your Mind
- Consider Concomitent Lesions(Other Peripheral Nerves)
- Train To Evaluate Tib Post.-Short Head Of Biceps-EHL-TFL

Iranian Congress OF Electrodiagnosis
IN
Peripheral Nerve Lesions
DEC.21.22/2006
Tehran-Iran

Foot Drop After Injection

- Sciatic Nerve injury
- Radiculopathy (L5)
- Diabetic Neuropathy
- In Addict Cases Sciatic Or DPN Palsy
- Wonderfuly A Case Had Bilateral Sciatic Nerve Injury (Diclofenac & Methocarbamol)
- Motor Neuron Disease
