# Supplementary material for: Meeting report on the first Iranian congress of electrodiagnosis in peripheral nerve lesions
Source: J Brachial Plex Peripher Nerve Inj. 2007 Apr 14;2:10. doi: 10.1186/1749-7221-2-10 (PMC1865540; doi:10.1186/1749-7221-2-10)
Supplement: Additional file 1 — Slides from the invited lectures and panel discussions. Compressed PDFs of 15 presentations and 2 panel discussions during the conference. [file 1749-7221-2-10-S1.zip › SURGICAL MANAGEMENT OF NERVE LESIONS.pdf]

Iranian Congress OF Electrodiagnosis
IN
Peripheral Nerve Lesions
DEC.21.22/2006
Tehran-Iran

Comparative Evaluation of Techniques used in Peripheral Nerve Repair

Dr. Gousheh
Shahid Beheshti University of Medical Sciences
Tehran, Iran

In this case, The distance between the two ends of median nerve in forearm region was so long that we thought nerve graft will not give any results. So we performed end to side anastomosis. After 1 ½ years, clinically, no motor or sensory results were observed.

In 1979, a war injury case of both right and left hand complete radial nerve paralysis was referred for reparative operation. I repaired the right radial nerve using the routine technique of the time by nerve grafting. Due to extreme exhaustion of performing many emergency operations that day, I repaired the left radial nerve by End-to-End anastomosis, which was easier.

I was surprised to see that the End-to-End repair recovered, but the hand repaired by nerve grafting was still paralyzed.

It was only 6 months after the recovery of End-to-End anastomosis that the grafted nerve showed signs of recovery.

In 1983, in the International Congress for Hand Surgery in Boston I reported 8 cases of End-to-End nerve repair, and in Buenos Aires in 1984 Microsurgery Congress, I reported 12 cases of nerve repair by End-to-End anastomosis.

To evaluate the results of End-to-End anastomosis with Nerve Grafting and End-to-Side anastomosis, we compared the results of 35 End-to-End, 35 Nerve Grafting, and 6 End-to-Side procedures in a retrospective study.

End-to-End: 35 Cases
Nerve Grafting: 35 Cases
End-to-Side: 6 Cases

This is a case of complete radial nerve palsy caused by an osteosynthesis operation by plate fixation 6 months after the paralysis.

Etiology of Cases

Patients age ranged 12 to 40 years (average 21 years).

The period between the nerve repair and the injury was 1 day to 9 months. Average of 3 months.

Nerve Graft<br>3 to 6 Cm Nerve Gap	End-to-End Anastomosis<br>1.5 to 3 Cm Nerve Gap
16 War Injury Damage	9 War Injury Damage
9 Arm Fracture (Iatrogenic cases)	5 Arm Fracture (Iatrogenic Cases)
10 Failed Primary Operations	21 Cases Cut by Sharp Objects (Iatrogenic)
Total 35 Cases	Total 35 Cases

Frequency distribution of the results of the End-to-End anastomosis of the radial nerve in wrist and fingers

Function \ Results		Excellent	Good	Fair	Bad	Total
Wrist Extension	Number of Patients	10	23	2	0	35
	Percent	28.6	65.7	5.7	0	100
Fingers Extension	Number of patients	8	20	4	3	35
	Percent	22.9	57.1	11.4	8.6	100

Iranian Congress OF Electrodiagnosis
IN
Peripheral Nerve Lesions
DEC.21.22/2006
Tehran-Iran

Frequency distribution of the results of the Nerve Grafting of the Radial nerve in wrist and fingers

Function \ Results		Excellent	Good	Fair	Bad	Total
Wrist Extension	Number of Patients	6	17	7	5	35
	Percent	17.1	48.6	20	14.3	100
Fingers Extension	Number of patients	4	14	7	10	35
	Percent	11.4	40	20	28.6	100

Iranian Congress OF Electrodiagnosis
IN
Peripheral Nerve Lesions
DEC.21.22/2006
Tehran-Iran

Comparison of Nerve Repair Results of End-to-End Anastomosis versus Nerve Grafting for Wrist Extension

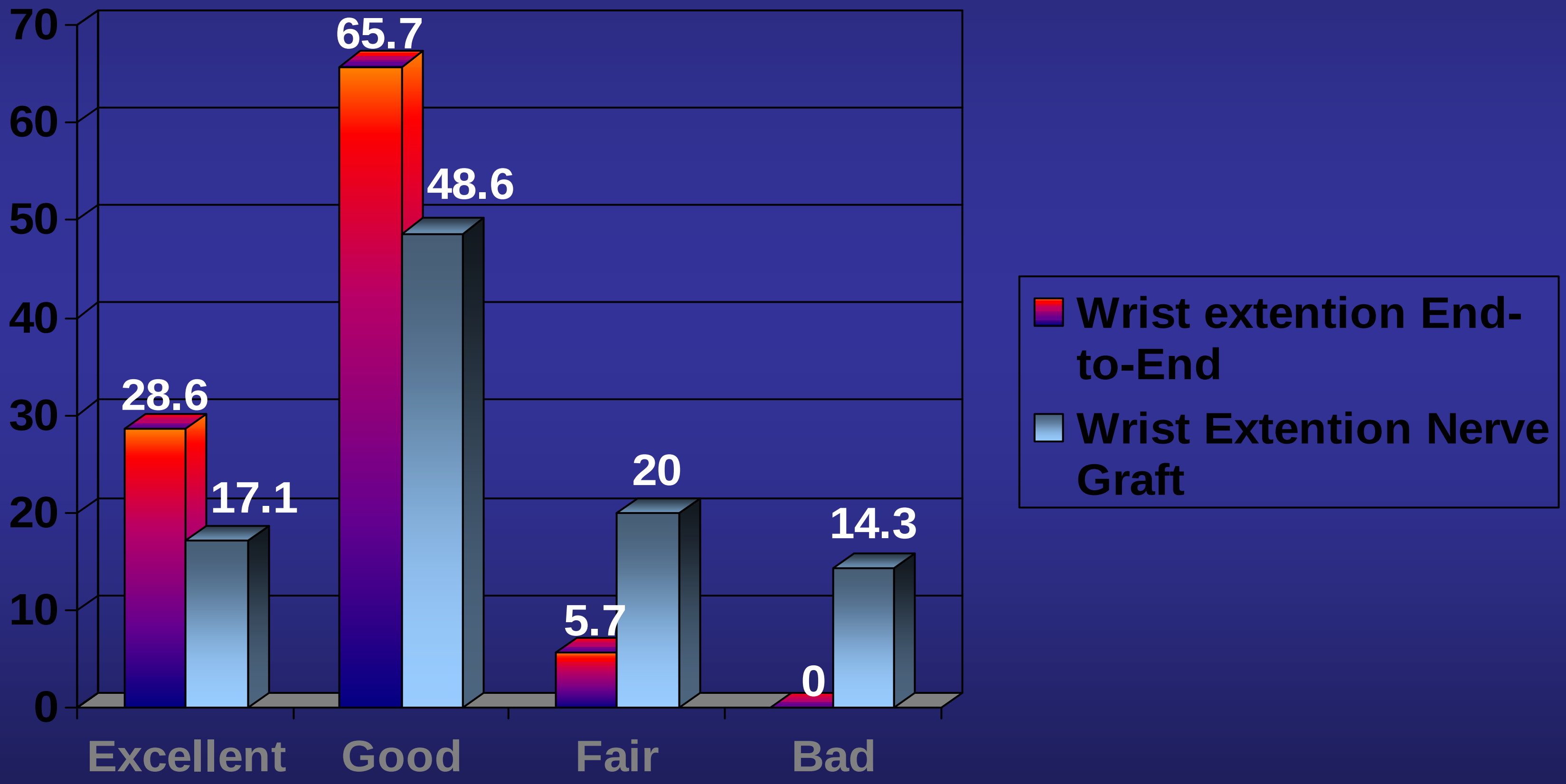

And as you see the results of end to end anastomosis (red one) are better than nerve grafting.

Iranian Congress OF Electrodiagnosis
IN
Peripheral Nerve Lesions
DEC.21.22/2006
Tehran-Iran

Comparison of Nerve Repair Results of End-to-End Anastomosis versus Nerve Grafting for Fingers Extension

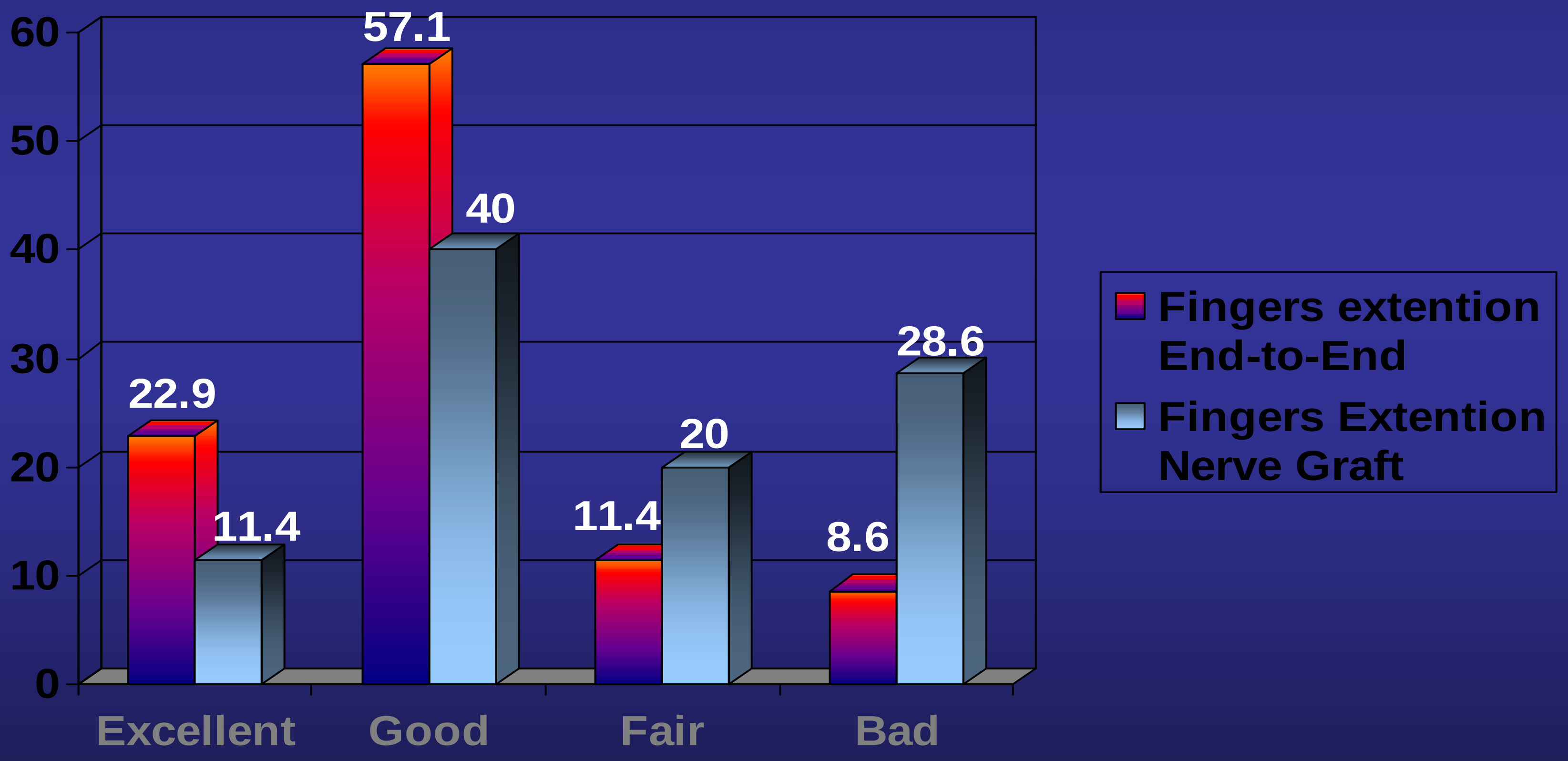

And as you see the results of end to end anasatomosis (red one) are better than nerve grafting.

The functional results of our work shows that end to end anastomosis is the Technique of choice if the ends of the nerve can be sutured with an 8/o nylon Since the motor recovery starts earlier, muscle atrophy is less, the atrophy recovers faster, and the functionality of the organ is reached in a shorter period of time.

For End-to-Side anastomosis, no sensory or motor nerve Recovery, was observed in any of the 6 operations we performed.

Also, our results indicate, the increase in the time interval between the injury and Nerve repair, reduces the chances of recovery.
