# Supplementary material for: Meeting report on the first Iranian congress of electrodiagnosis in peripheral nerve lesions
Source: J Brachial Plex Peripher Nerve Inj. 2007 Apr 14;2:10. doi: 10.1186/1749-7221-2-10 (PMC1865540; doi:10.1186/1749-7221-2-10)
Supplement: Additional file 1 — Slides from the invited lectures and panel discussions. Compressed PDFs of 15 presentations and 2 panel discussions during the conference. [file 1749-7221-2-10-S1.zip › TOS.pdf]

Iranian Congress OF Electrodiagnosis
IN
Peripheral Nerve Lesions
DEC.21.22/2006
Tehran-Iran

ELECTRODIAGNOSIS IN T.O.S.

KAMIAR AKRAMI M.D.

TEHRAN UNIVERSITY OF MEDICAL SCIENCES

T.O.S.

- Complex symptoms caused by compression

Of the brachial, or

the axillary-subclavian

artery or vein as

they exit the chest.

Sites of compression

1. Interscalene space or triangle
2. Costoclavicular space
3. Subpectoralis minor space

Anatomic Variations

- Cervical rib
- Elongated C7 transverse process
- Fibrous bands
- Hypertrophy of anterior scalene m.
- Passage of brachial plexus through the substance of scalenous m.
- Variations in scalen triangle

TOS

- ***Until 1920***

Vascular condition,

(compression of subclavian artery by a congenital anomaly)

- ***Today***

Neurogenic condition, caused by anatomic predisposition & neck trauma

TOS

1. **Neurogenic TOS** ***(true neurogenic TOS)*** with typical findings of brachial plexus compression
2. **Vascular TOS** (compression of subclavian vessels either artery or vein)
3. **Nonspecific type TOS** ***(disputed TOS)***

Clinical presentation
true Neurogenic TOS

- Young female with a long neck & drooping shoulder
- Relatively **painless**
- Gilliat- Summer hand
- Sensory loss (ulnar aspect of
  hand & forearm)
- Discomfort provoked by repetitive use of extremity with over head activity

Nonspecific type TOS
(disputed TOS)

- Young and middle age women
- Intermittent ***pain*** or stiffness in the hand & forearm
- Subjective complaints of weakness in the affected limb
- ***Neck pain***
- Supraclavicular tenderness
- ***headaches***

Gilliat et al. 1978

■ Needle exam	■ Always abnormal
APB	↓ IP to a single unit
FDIO	↓ IP to a single unit
■ CMAP amplitude	
APB	Low or very low
FDIO	Low or very low
■ SNAP amplitude	
Median	Normal
Ulnar	Comparatively low to absent
MABCN	***Not tested***

CTS & TOS

- Chronic entrapment neuropathy
- Usually displays a clinical pattern of transient complaints & absence of amyotrophy & hyposthesia for a long time

- Phalen (1966): amyotrophy and hyposthesia in 50 & 80 % of patients ; nowadays , they are found in 5 & 15% of patients.

Conduction across the brachial plexus

- Combined supraclavicular & axillary stimulation while recording from hypothenar eminence

- The results could not be reproduced by other investigators

SEP of median & ulnar & also dermatomal SEP

- Parameter in use was ulnar ***amplitude*** recorded from supraclavicular fossa.
- Comparatively small amplitude in normal persons & subject to variation with arm position.
- SEP doesn't add much to the simple & routine nerve conduction studies.

Axillary F loop latencies :

- F wave conduction across the thoracic outlet region was performed

F Wave technique for AFCL of the ulnar and median nerves

A. Stimulate at the wrist and record M latency.
B. Stimulate at the wrist and record F latency as described above.
C. Measure from the sternal notch to the axilla a distance of 25 cm and stimulate here to record an M latency (axilla site).
D. Calculate the AFCL from the formula AFCL = (Mw + Fw)–2 x M axilla

F wave testing with the arms in provocative position

- Provocative position : position that would generally bring about the symptoms.
- Provocative positions are different for subjects .
- F waves obtained in provocative position for approximately 1 min revealed delays of the minimal F latency in the median , ulnar or both in the symptomatic arm but no change in the symptomatic arm.

Am.J.Med.Rehabil. Vol. 82. No.2 . 2003

Stimulation of C8-T1 spinal nerve fibers

75 mm monopolar needle , through paraspinalis m., reference electrode on midthoracic skin. Stimulation intensity of 50-100 mA & 0.7-1 ms duration , recording response from APB & ADM.

MABCN
medial antebrachial cutaneous nerve1

- Lowest branch of brachial plexus
- Sensory fibers arise from T1 and accessorily C8 root.

- Travel through lower trunk & medial cord.

- Mild lesions of the lower brachial plexus involving T1 component of the lower plexus do not cause either amyotrophy or finger sensory impairment & could be electrodiagnosed with the sole finding of abnormal MABCN sensory action potential.

- MABCN predominantly carries sensory fibers from the T1 root wich is the first to suffer from angulation on a cervical rib or fibrous band .

MABCN
medial antebrachial cutaneous nerve 2

- MABCN pierces the deep fascia 10 cm above medial epicondyle with basilic vein and divides into an anterior and a minor posterior branch.
- Provides sensory supply of medial aspect of the forearm and olecranon.

NCS OF MABCN

- ***Antidromic study*** of the anterior branch of the MABCN with bipolar stimulation 1 to 3 cm above and before the medial epicondyle and bipolar recording 8-12 cm distally on anteromedial aspect of the forearm.
- ***Orthodromic method*** : stimulation and recording sites are reversed compared to antidromic technique.

- When only one test is done (ortho or antidromic) a ratio greater than 2.0 is required to assess a unilateral abnormal MABCN.
- When the ratio is > 1.66 & < 2 with one method , the other method should be used as a confirmation. If the ratio is again > 1.66 , the MABCN is deemed abnormal.
